# Evolution of Phage Tail Sheath Protein

**DOI:** 10.3390/v14061148

**Published:** 2022-05-26

**Authors:** Peter Evseev, Mikhail Shneider, Konstantin Miroshnikov

**Affiliations:** Shemyakin-Ovchinnikov Institute of Bioorganic Chemistry, Russian Academy of Sciences, Miklukho-Maklaya Str., 16/10, 117997 Moscow, Russia; mm_shn@mail.ru

**Keywords:** sheath protein, tail contraction, phage tail assembly

## Abstract

Sheath proteins comprise a part of the contractile molecular machinery present in bacteriophages with myoviral morphology, contractile injection systems, and the type VI secretion system (T6SS) found in many Gram-negative bacteria. Previous research on sheath proteins has demonstrated that they share common structural features, even though they vary in their size and primary sequence. In this study, 112 contractile phage tail sheath proteins (TShP) representing different groups of bacteriophages and archaeal viruses with myoviral morphology have been modelled with the novel machine learning software, AlphaFold 2. The obtained structures have been analysed and conserved and variable protein parts and domains have been identified. The common core domain of all studied sheath proteins, including viral and T6SS proteins, comprised both N-terminal and C-terminal parts, whereas the other parts consisted of one or several moderately conserved domains, presumably added during phage evolution. The conserved core appears to be responsible for interaction with the tail tube protein and assembly of the phage tail. Additional domains may have evolved to maintain the stability of the virion or for adsorption to the host cell. Evolutionary relations between TShPs representing distinct viral groups have been proposed using a phylogenetic analysis based on overall structural similarity and other analyses.

## 1. Introduction

Tail sheath proteins (TShP) have a particular role in the structural biology of phages as a molecular engine for viral infection. The first object in the study of sheath proteins was gp18 from the classic phage T4. Unfortunately, it was not the best choice, because the recombinant protein tended to polymerise, forming polysheaths. Therefore, the structure was determined for a protease-resistant fragment (amino acids 83–365) and a deletional mutant of this protein (residues 1–510) [1]. Thus, the fine details of tail sheath contraction remained understudied. Meanwhile, it was shown that less complicated sheath proteins from prophages do not form polymeric structures, and their crystal structures could be revealed (3HXL and 3LML). X-ray analysis of the protease-resistant fragment of phage phiKZ TShP allowed scientists to compare the structures of sheath proteins from distant phages and revealed a common fold in this type of protein [2].

Development of cryo-electron microscopy enabled the reconstruction of whole particles of many bacteriophages with near to atomic resolution. Therefore, it was possible to work with the structures of a number of proteins from different phages and bacteriocins (T4, 812, A511, anti-feeding prophage, PVC). Some contractile systems have been shown to be organised from several sheath proteins combined to form complex structures with specific intermittence of layers [3,4]. The tail sheaths of some phages have a complicated morphology with sheath proteins encrusted by proteinaceous fibres (AR9, PBS1 [5]).

Natural contractile injection systems (CISs) are numerous and diverse. They can be further subdivided into: (i) those mediating bacterial cell–cell interactions, such as type VI secretion systems (T6SSs), and (ii) extracellular CISs (eCISs). All these nanomachines possess their own sheath proteins, which are often different from phage-originated ones, corresponding to their function. For instance, T6SS sheaths are formed by two proteins, TssB1/TssC1 (VipA/VipB). These sheaths can undergo several cycles of assembly, contraction and disassembly [6,7]. The diversity of eCISs (including bacteriocins) is also very high [8] and variations in the structure of their sheath proteins have yet to be investigated.

Recently, several modelling programs applying efficient machine-learning and deep neural-network algorithms to protein tertiary structure modelling have been developed [9,10,11]. The 14th Critical Assessment of protein Structure Prediction (CASP14) competition showed that the neural network-based software AlphaFold demonstrated “accuracy competitive with experimental structures in a majority of cases and greatly outperforming other methods”; the best-predicted 95% of residues in AlphaFold models had a median alpha carbon RMSD of 0.96 Å to experimental models [9]. The authors’ own analyses have also achieved high scores for the assessment of predicted models, which have been significantly higher than those of homologous modelling [12,13]. The AlphaFold structure predictions cover part of catalogued proteins in the UniProt [14] and the number of models contained in the AlphaFold Protein Structure Database currently stands at about one million, and this figure continues to increase [15]. The high level of accuracy of AlphaFold’s structure modelling has inspired its use in evolutionary analysis.

The current paper introduces an analysis of the structural evolution of tail sheath proteins belonging to different phage groups. First, an analysis was made of the common structural features of sheath proteins determined experimentally. Then, the representative phage sheath proteins were modelled using AlphaFold 2, novel machine learning software. Next, the structures obtained were analysed to enable possible patterns of evolutionary pathways to be proposed using phylogenetic analysis based on overall structural similarity and regular phylogenetic analysis of conserved phage proteins. This paper will also present a discussion of the driving forces for phage sheath protein evolution and discuss the applicability of regular phylogenetic analyses.

Several reviews discussing structural phage proteins were published recently [16,17,18,19,20,21,22]. Unfortunately, evolutionary issues were not elucidated except for [22]. The purpose of this study was to find patterns in the evolution of phage tail sheath protein and to suggest a hypothesis for the evolution of the structural architecture of phage tail sheath proteins.

## 2. Materials and Methods

### 2.1. Data Acquisition and Annotation

The protein tertiary structures were downloaded from the Research Collaboratory for Structural Bioinformatics Protein Data Bank (RCSB PDB) [23]. The genomes were downloaded from the NCBI Genome database [24]. Phage annotation was checked and re-annotated, if needed. Re-annotation was conducted using Glimmer 3.0 [25] and Prokka 1.14.5 [26]. Protein functions were assigned with the assistance of a BLAST homology search [27], HHM-HHM-motif comparison using the HHpred server [28], InterProScan 5 [29], protein fold recognition server Phyre2 [30], and protein structure modelling with AlphaFold 2.1 [9], with subsequent superimposition executed with known structures using Pymol 2.4.1 [31].

### 2.2. Protein Sequence Alignment and Phylogeny

Annotated genes were extracted and translated. Primary sequence alignments were made with Clustal Omega 1.2.4 [32] and MAFFT 7.48 with default settings using the L-INS-i algorithm [33]. The phylogenetic trees based on these alignments were constructed using FastTree 2 [34] with default settings and using RAxML-NG [35] integrated with a raxmlGUI 2.0.7 graphic interface [36] with (--model LG + G --bs-metric tbe --tree rand{10} --bs-trees 1000) settings. The best protein model was found with ModelTest-NG [37] integrated into raxmlGUI. The robustness of the RAxML trees was assessed by bootstrapping. The trees were constructed using the iTOL server [38].

### 2.3. Protein Tertiary Structure Modelling, Visualisation and In Silico Analysis

Sheath protein structural modelling was carried out using AlphaFold 2.1.1 [9] with full databases running on a local machine (16-core AMD 5950x processor and Nvidia GeForce RTX 3090 video card with 24 GB memory). The models were visualised and superimposed in Pymol 2.4.1 [31]. Multiple structure alignments were obtained using an mTM-align server [39] or a local machine [40]. Structure-based phylogenetic trees were plotted using an mTM-align server [39] or an mTM-align local version and neighbour-joining tree clustering implemented in the PHYLIP Phylogeny Inference Package 3.6 [41]. Protein topology graphs were plotted using the Protein Topology Graph Library server (PTGLweb) [42].

## 3. Results

### 3.1. Sheath Proteins in the RCSB Protein Bank Database

**Figure 1:**
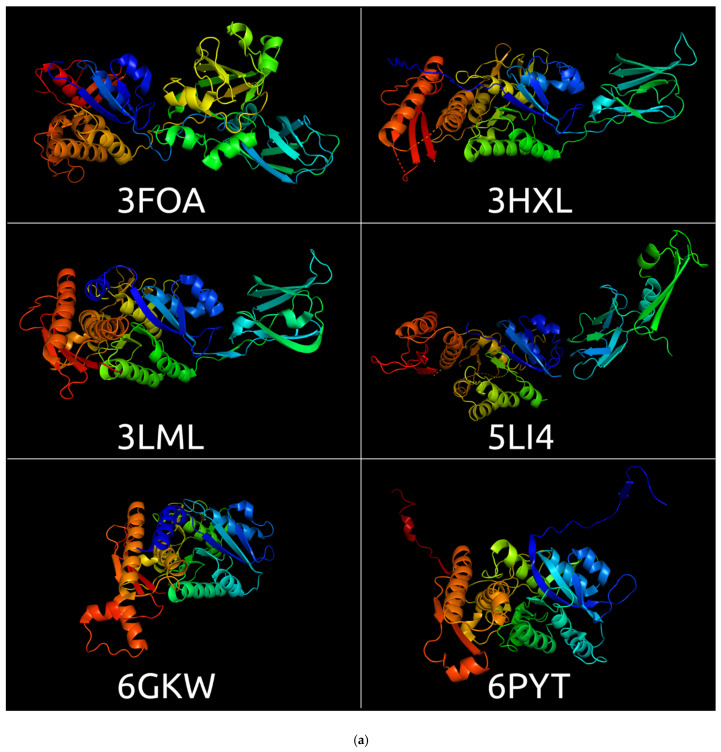

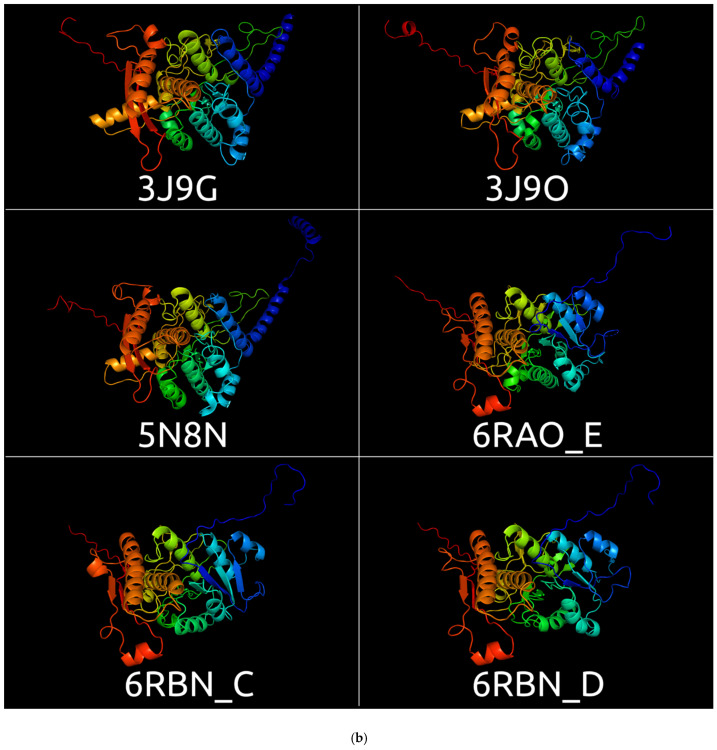
RCSB Protein Bank Database structures depicted with Pymol. (**a**) 3FOA, crystal structure of the bacteriophage T4 tail sheath protein, deletion mutant gp18M; 3HXL, crystal structure of the sheath tail protein (DSY3957) from *Desulfitobacterium hafniense*; 3LML, crystal structure of the sheath tail protein Lin1278 from *Listeria innocua*; 5LI4, bacteriophage phi812K1-420 tail sheath protein after contraction; 6GKW, crystal structure of the R-type bacteriocin sheath protein CD1363 from *Clostridium difficile* in the pre-assembled state; 6PYT, cryoEM structure of precontracted pyocin R2 trunk from *Pseudomonas aeruginosa*. (**b**) 3J9G, sheath protein (VipB) from the type VI secretion system of *Vibrio cholerae*; 3J9O, sheath protein (IglB) from the type VI secretion system of *Francisella tularensis* subsp. *novicida*; 5N8N, sheath protein (TssC) from the type VI secretion system of *Pseudomonas aeruginosa*; 6RAO_E, 6RBN_C, 6RBN_D, three sheath proteins of the anti-feeding prophage (AFP) of *Serratia entomophila*. The models are coloured based on a rainbow gradient scheme, where the N-terminus of the polypeptide chain is coloured blue, and the C-terminus is coloured red.

There are several records in the RCSB Protein Bank Database [23,43] regarding the structures of sheath proteins determined with a comparatively high resolution of 1.8–4.2 Å (Table 1, Figure 1) that belong to phages, prophage regions, bacteriocins, the anti-feeding prophage system (AFP), and the type VI secretion system evolutionarily related with phage TShPs [44,45]. In addition, several crystal structures and reconstructions have been determined with a lower resolution.

The sheath proteins of *Serratia entomophila* AFP and *Photorhabdus asymbiotica* CIS are comprised of three proteins encoded by three adjacent genes located in the contractile molecular machine genes cluster. The alignment of the amino acid sequences for sheath proteins encoded by different genes indicate their relatedness with one another and show a well-marked homology between the proteins belonging to these two species.

### 3.2. Positioning of the Conserved Core in Experimentally Determined TShPs

**Figure 2:**
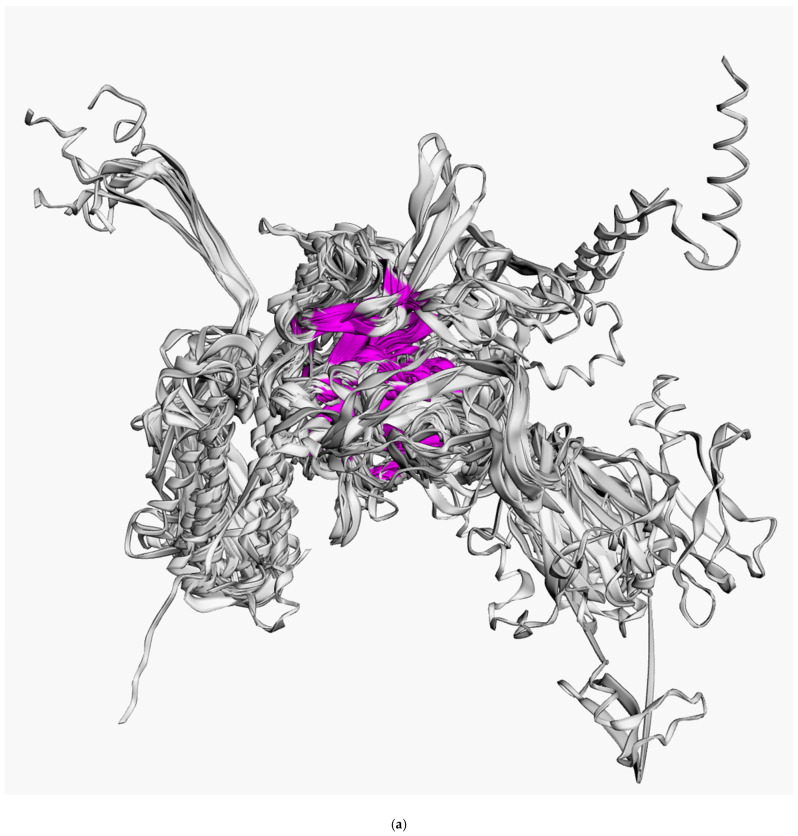

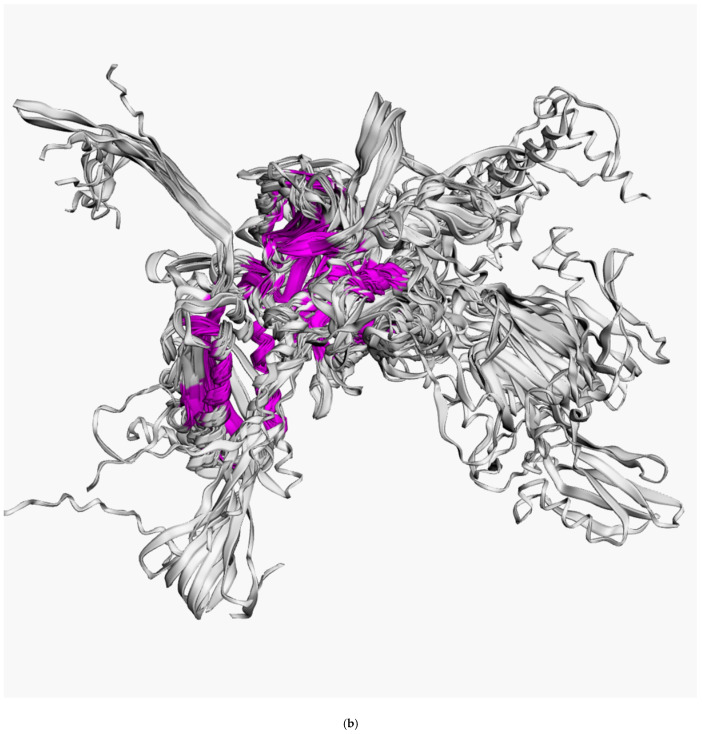

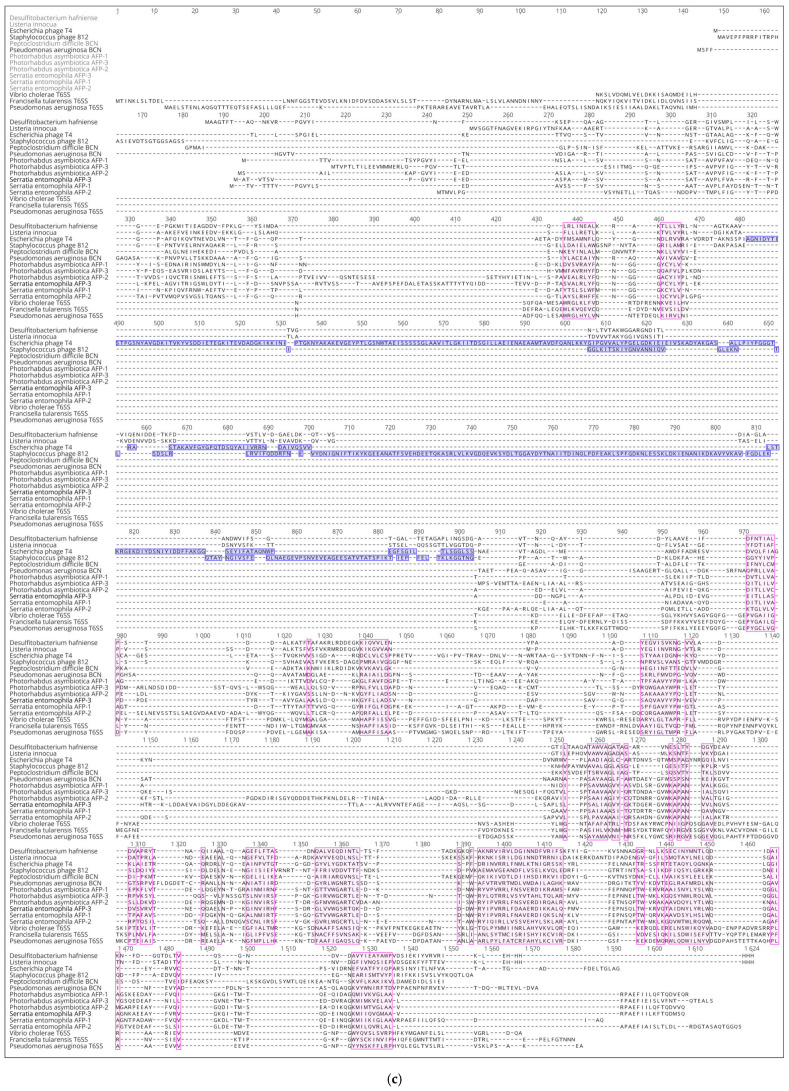

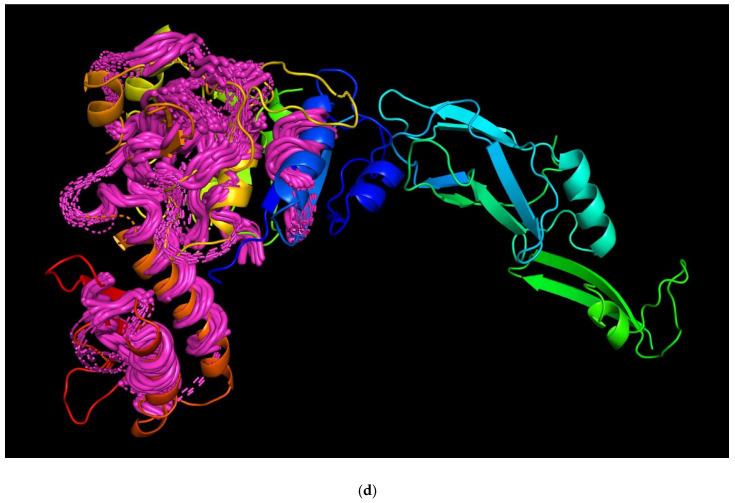
(**a**) Visualisation of the structural alignment made with mTM-align for fifteen experimentally determined sheath proteins (deletion mutant of *Escherichia* phage T4 TShP; *Desulfitobacterium hafniense* prophage TShP; *Listeria innocua* prophage TShP; *Staphylococcus* phage 812 TShP; R-type bacteriocin sheath protein from *Peptoclostridium difficile*; pyocin R2 sheath protein from *Pseudomonas aeruginosa*; sheath proteins of the type VI secretion system from *Francisella tularensis* subsp. *novicida*, *Pseudomonas aeruginosa*, and *Vibrio cholerae*; anti-feeding prophage sheaths from *Serratia entomophila* and *Photorhabdus asymbiotica*). The proteins are depicted as ribbons. The parts with a maximum pairwise residue distance of less than 4 Å are coloured magenta. (**b**) Visualisation of the structural alignment of the fifteen modelled sheath proteins obtained by the translation of genes encoding the proteins used for the experimentally determined structures listed in Figure 2a. (**c**) Structural alignment of the fifteen modelled sheath proteins obtained by the translation of genes encoding the proteins used for the experimentally determined structures listed in Figure 2a. Columns in magenta have a maximum pairwise residue distance of less than 4Å. The insertions interrupting the conserved domains of phages T4 and 812 are coloured blue. (**d**) The 3D-model of the TShP of *Staphylococcus* phage 812, coloured according to a rainbow gradient scheme, where the N-terminus of the polypeptide chain is coloured blue, the C-terminus is coloured red, and the model superimposed with the “common core” of the experimentally determined sheath is coloured magenta.

**Figure 3:**
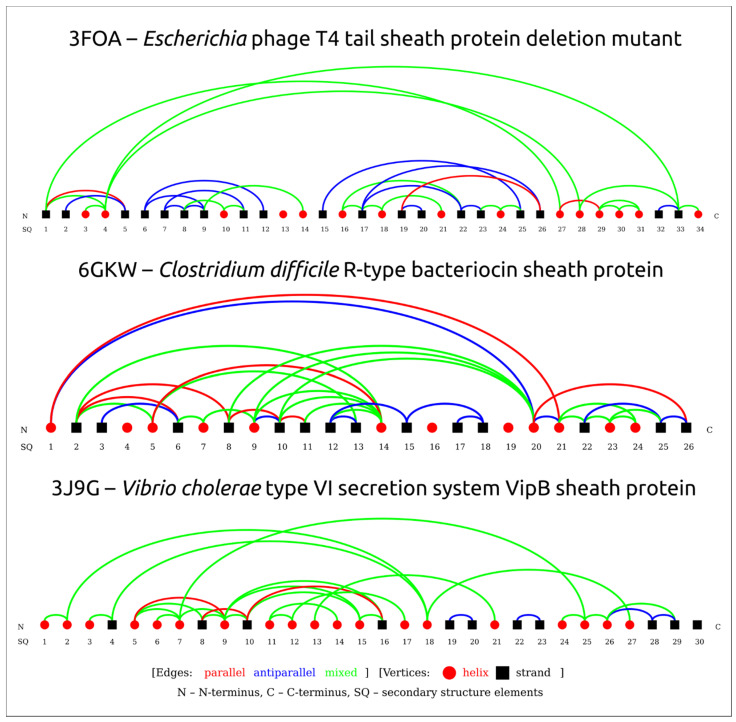
Protein topology graphs based on PDB structures constructed using the PTGL database for secondary structure-based protein topologies. Structural elements are depicted as geometric figures according to the legends. Connections between structural elements are shown as lines coloured according to the legends.

Superimposition of the structures depicted in Figure 1 indicated distinct structural similarities for sheath proteins belonging to phage tails, T6SS, and the extracellular contractile injection system. Structural alignment of the experimentally acquired structures clearly showed the presence of a conserved core shared by all aligned proteins (Figure 2a). Several determined structures lacked some residues but structural alignment using the AlphaFold 2 models showed the conserved core to a fuller extent (Figure 2b). In particular, the conserved part of the protein from *Escherichia* phage T4 and *Staphylococcus* phage 812 is interrupted by long insertions (Figure 2c) and includes residues located in both the N-terminal and C-terminal parts. Remote contacts between the different regions illustrated in protein topology graphs demonstrate mixed connections between the N-terminal and C-terminal parts of the experimentally determined structure of the TShP deletion mutant 3FOA and mostly antiparallel connections within the central regions of the strands (Figure 3).

**Figure 4:**
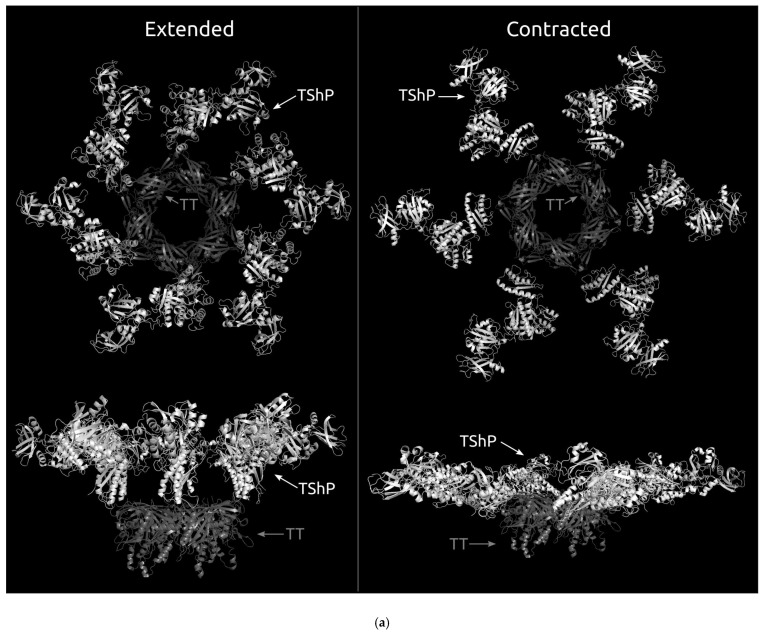

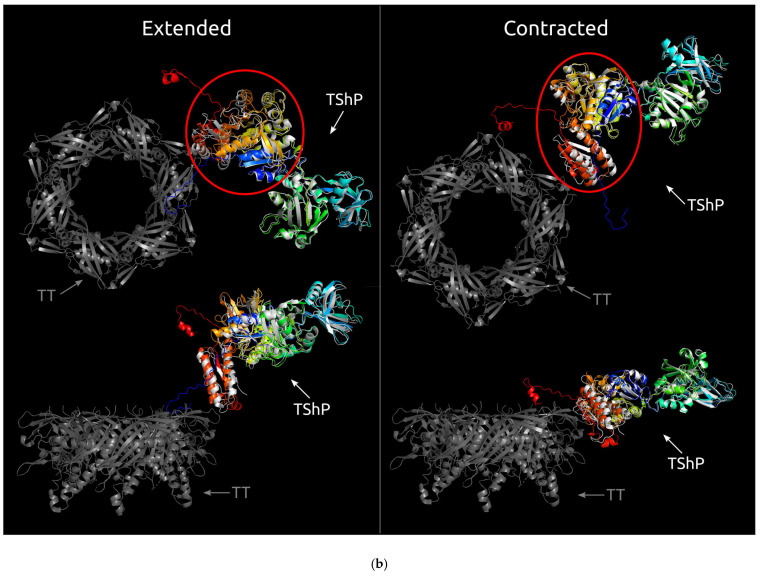
(**a**) Structure of the tail tube hexamer (coloured grey) and the model of the tail sheath protein (coloured white) fitted into the cryo-EM reconstruction of the T4 tail [56] in the extended (3J2M) and contracted (3J2N) states. (**b**) The same as Figure 4a but superimposed with the AlphaFold 2 model of the T4 tail sheath. The AlphaFold 2 model is coloured based on a rainbow gradient scheme, where the N-terminus of the polypeptide chain is coloured blue, and the C-terminus is coloured red. The conserved core is circled red. TT, tail tube proteins; TshP, tail sheath proteins.

To clarify the position of the conserved common core in the phage tail, the previously published results [56] for the cryo-EM reconstruction of the extended (3J2M) and contracted (3J2N) tail of phage T4 were used (Figure 4a). The original reconstruction contains the fitted model of the tail sheath protein built on the basis of the experimentally determined structure and the results of structure modelling. The superimposed AlphaFold 2 model of the phage T4 tail sheath protein was also used (Figure 4b). This model is similar to the original model used.

A visual analysis of the models obtained (Figure 4b) shows that the conserved core is closer to the tail tube than the other parts of the protein. The N-terminus is located more distantly from the tail tube proteins than the C-terminus, but the domains outside of the common core are placed even farther from the tail tube. This indicates that interactions may exist between the tail tube and tail sheath proteins, and the common core part of the sheath protein may be important for correct phage tail assembly. The cryo-EM reconstruction of *Staphylococcus* phage 812 indicates a similar layout [48].

### 3.3. Choosing Representative Sequences for Modelling

At the beginning of 2022, the classification of bacteriophages approved by the International Committee on Taxonomy of Viruses (ICTV) [57] included four families of phages with myoviral morphology, namely, *Myoviridae*, *Ackermannviridae*, *Chaseviridae*, and *Herelleviridae*. In addition, a gene encoding a tail sheath protein with myoviral morphology was found in the genome of *Paenibacillus* phage Lily [58], comprising the singleton *Lilyvirus* genus, not assigned to any phage family [57]. This phage was first reported as a siphovirus, but its genome shows a high level of similarity with *Paenibacillus* phage ERIC V (81.6% average nucleotide identity, according to orthoANI calculations) [59]. The latter is classified as a member of the *Myoviridae* family. *Ackermannviridae*, *Chaseviridae*, and *Herelleviridae* groups were delineated from the *Myoviridae* family. The re-evaluation of bacteriophage taxonomy continues. Currently, the *Myoviridae* group seems to be the most diverse. This diversity can be explained by the fact that, at the present time, the formation of new taxa is based on genomic/proteomic features, whereas the contractile tail, a hallmark of myoviruses, is a morphological property. A recent 2021 ICTV proposal (not yet ratified) suggests abolishing the definition of *Myoviridae* as a virus family, leaving a taxonomical gap between class *Caudoviricetes* and subfamilies/separate genera for describing phages with myoviral morphology. Nevertheless, the contractile tail is an important structural and functional feature, which especially concerns the subject of this discussion. Therefore, the *Myoviridae* term will be retained for the purposes of the current paper. An analysis of the alignments and HMM-HMM motif comparisons have indicated that the TShPs of phages belonging to the *Ackermannviridae, Chaseviridae*, and *Herelleviridae* families possess conspicuous similarities to one another within those groups, whereas the TShPs of phages belonging to the other myoviruses are the most diverse. At the beginning of January 2022, the GenBank phage database contained 278 entries attributed as *Ackermannviridae*, 34 entries attributed as *Chaseviridae*, 509 entries attributed as *Herelleviridae*, and 5723 entries attributed as *Myoviridae*.

Special attention has been paid to archaeal viruses because of their great significance for evolutionary biology. Many archaeal viruses are morphologically indistinguishable from tailed bacteriophages [20,60,61], and a genomic analysis of archaeal myoviruses has indicated the presence of tail sheath proteins reminiscent of some bacterial myophages.

In January 2022, the GenBank phage database contained 43 complete genomes for archaeal myoviruses. The genomes of the viruses listed below encode distinguishable putative tail sheath proteins:

1. *Haloarcula* phages of *Haloferacalesvirus* genus: Ten complete genomes (HCTV-6, -7, -8, -9, -10, -11, -15 and HJTV-1, -2, -3) possess similar genome organisation and length. The predicted TShPs possess 431 to 438 amino acid (aa) residues. HCTV-6 and HCTV-15 are identical and differ in their primary sequence from eight other TSPs (% identity is about 45–47%). The latter proteins are very similar, or identical, showing a pairwise identity of 84% and higher.
2. *Halobacterium* phages of *Myohalovirus* genus: two complete genomes (phiH and ChaoS9) contain two TSPs of about 430 aa length that have 52% identity and identical HHM-HHM motif comparison results obtained with HHpred [28].
3. *Haloferax* phage HF1 of *Haloferacalesvirus* genus: the HF1 tail sheath protein amino acid sequence is similar to the TShPs of *Haloarcula* phages HCTV-7, -8, -9, -10, -11, and HJTV 1, -2, -3, and has an identity to *Haloarcula* phages of about 90%.
4. *Halorubrum* phages of *Haloferacalesvirus* genus: 28 genomes encode the TShPs of about 430 aa lengths. Of these, 23 TShP sequences are very similar to one another (83–99%). They belong to *Halobrum* phage HF2; *Halorubrum* Tailed Viruses 5 and 8; phages Hardycor2; *Halorubrum* viruses HRTV-9, -10, -13, -14, -15, -16, -17, -18, -19, -20, -21, -22, -23, -24, -26; Halorubrum virus HSTV-4; Serpecor1; and VOLN27B. Tail sheath proteins from *Halovirus* HSTV-2, *Halorubrum* Tailed Virus 7, and *Halorubrum* viruses HRTV-2 and HRTV-11 have 97–98% identity with one another and less than 50% identity with all other *Halobrum* phages, and constitute another group of *Halorubrum* phage TShPs. The TShPs of phages HRTV-25 and HRTV-27 show less than 40–50% primary sequence identity with one another and all other *Halobrum* TShPs.
5. *Natrialba* phage φCh1 of *Myohalovirus* genus: a TShP was revealed by a BLAST and HMM search. The protein is 426 aa in length and shows about 40% identity with *Halobacterium* phages.

Representatives from all of the groups listed above were used for modelling.

A BLAST search using the GenBank Bacterial database, containing archaeal and bacterial chromosomes and plasmid sequences, revealed that putative TShPs were encoded in *Natronorubrum bangense* strain JCM10635, *Methanolacinia petrolearia* DSM 11571, and other *Euryarchaeota*. The primary sequences of archaeal TShPs are often distant from known bacterial myovirus TShPs. Apparent homologs of archaeal TShPs have, however, been found in *Pseudomonas* phages belonging to the genus *Otagovirus* (for example, phage PPSC2), plasmids of *Clostridium baratii* str. Sullivan, and other bacterial plasmids and chromosomes.

Interestingly, homologs of sheath proteins can also be found in archaeal genomes that are being part of the Asgard group *Lokiarchaeota*, *Thorarchaeota*, *Crenarchaeota*, *Bathyarchaeota*, and *Pacearchaeota*. Functional assignments for these homologous proteins were predicted by a BLAST search and HHM-HHM motif comparison.

After a preliminary analysis, about 2000 TShP sequences extracted from annotated or re-annotated viral and prokaryotic genomes, both predicted and experimentally found, were used for fast phylogenetic tree construction by means of FastTree [34]. Some phage genomes encoded two copies of tail sheath proteins. It has been shown for several Jumbo phages that they also arose by gene duplication [62]. In those cases where the phage genome encoded more than one TShP, only one was used for further analysis. A total of 109 phage sequences representing different clades of the tree, phage hosts, and taxa were selected. This included the various representatives of archaeal and bacterial *Myoviridae*, *Ackermannviridae*, *Chaseviridae*, and *Herelleviridae* families, and genus *Lilyvirus*. Archaeal proteins were used for a BLAST search of the archaeal and bacterial GenBank database to find homologs in archaeal and bacterial genomes. Three putative TShP sequences were added from the list of Jumbo phages predicted by the metagenome analysis in [63]. Sequences for experimentally found sheath proteins were also used to search for homologs in the genomes of bacteria and archaea.

In addition, phages belonging to the recently established *Schitoviridae* family of N4-like phages [64] possess a receptor known as the “non-contractile tail sheath protein” [65]. Two of these proteins, from phages *Escherichia* phage AlfredRasser (subfamily *Enquatrovirinae*, genus *Enquatrovirus*) and *Delftia* phage RG-2014 (genus *Dendoorenvirus*), were taken for further analysis. The sequences shown in Figure 2c and experimentally determined earlier were also taken for modelling using translated genes extracted from the corresponding genomes.

The total number of selected sequences was 155. This included 114 phage tail sheath proteins (112 contractile and 2 non-contractile), 25 sheath proteins homologous to archaeal tail sheaths from archaeal and bacterial chromosomes and plasmids, 8 sheath proteins from the type VI secretory system, 6 proteins from the extracellular contractile injection system (anti-feeding prophage), and 2 sequences for sheath proteins from bacteriocins (pyocin and diffocin). The functional assignments of all selected proteins were confirmed with a BLAST search and HHM-HHM motif comparison.

### 3.4. Modelling and General Structural Analysis of Representative Sheath Proteins

**Figure 5:**
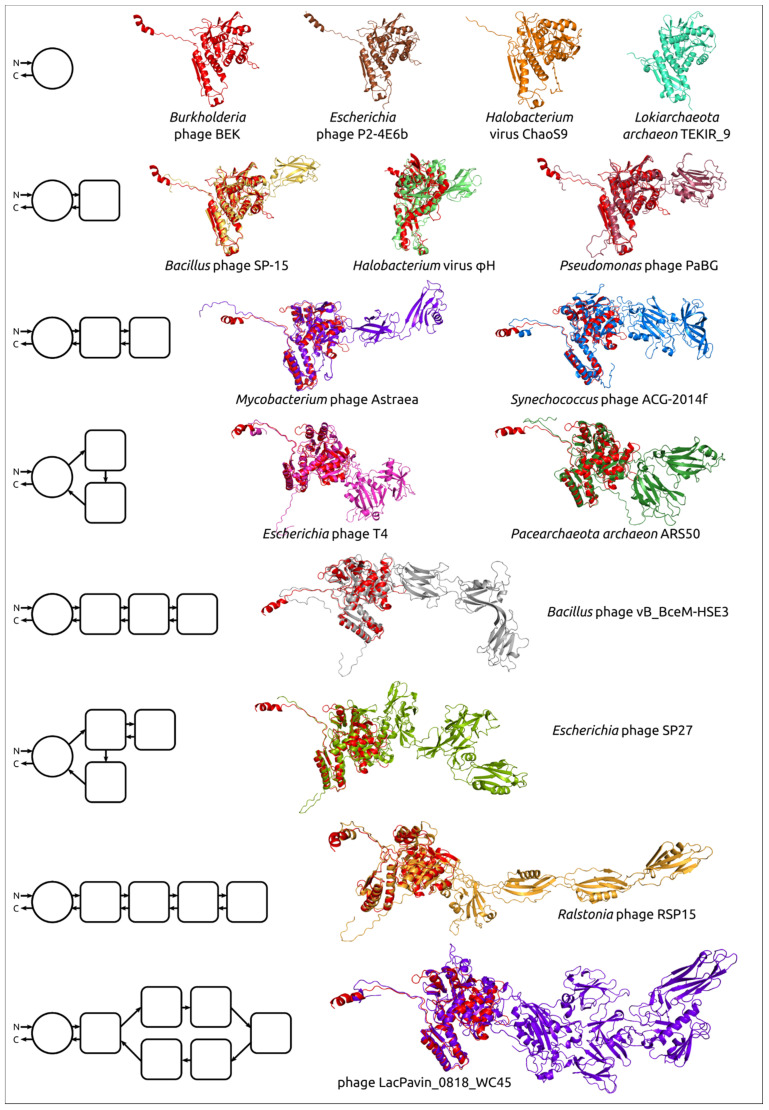
Examples of the structural architecture for the modelled contractile phage sheath proteins. The TShPs consisting of two and more domains are superimposed with the modelled structure of *Burkholderia* phage BEK tail sheath protein, depicted in the red colour. The schemes on the left show the structural architecture of proteins. The main domain is depicted as a circle, and the additional domains are represented as squares with rounded corners. The direction of the polypeptide chain from the N- to C-termini is shown with arrows.

Visual analysis of the modelled contractile sheath proteins revealed the different structural architecture of the models. All shared the conserved domain, composed of the N-terminal and C-terminal parts, and some had additional domains (from the point of view of a domain as a compact structure [66]). In a few cases, the modelled structures did not have clearly distinguishable domains, but in most cases, it was possible to estimate the number of domains. As expected, the so-called “non-contractile” receptor-binding “tail sheath protein” of *Schitoviridae* phages had a completely different fold and was not analysed further. Examples of the structural architecture for the modelled contractile phage sheath proteins are shown in Figure 5. The PDB files of all best-ranked modelled structures and FASTA sequences are included in the Supplementary Data (Supplementary Files S1 and S2).

#### 3.4.1. One-Domain Contractile Sheath Proteins (Type 1)

**Figure 6:**
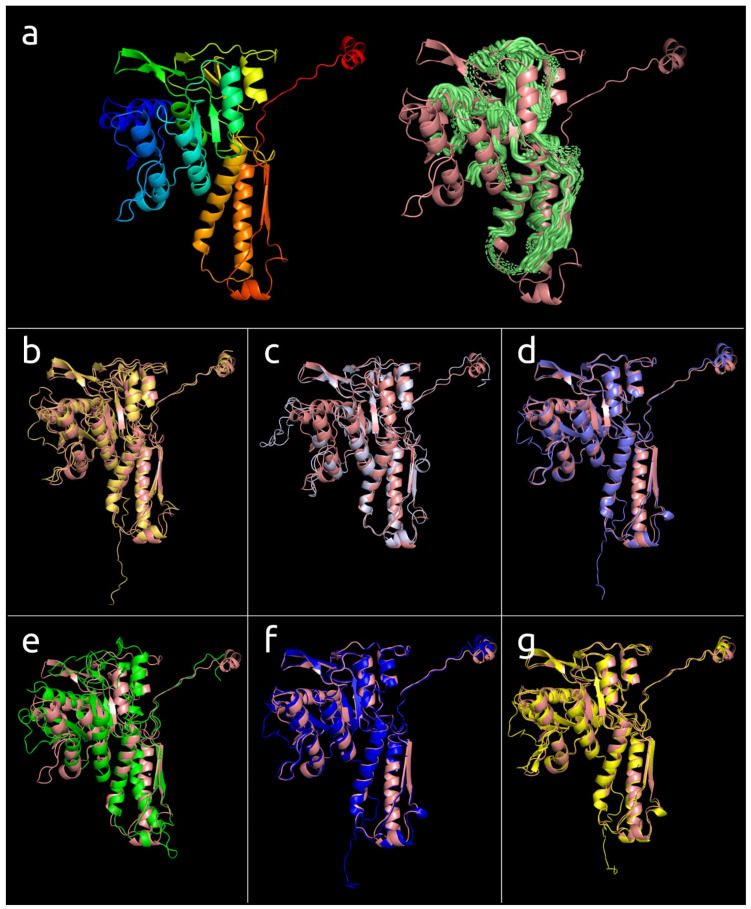
(**a**) The 3D-model of the TShP of *Burkholderia* phage BEK (left) coloured according to a rainbow gradient scheme, where the N-terminus of the polypeptide chain is coloured blue, the C-terminus is coloured red, and the model superimposed with the “common core” of the experimentally determined sheath proteins shown in Figure 2. (**b**) The model of the TShP of *Halomonas* phage HAP1 (yellow orange) superimposed with *Burkholderia* phage BEK TShP (salmon). (**c**) The model of putative sheath protein found in the genome assembly attributed as *Candidatus Bathyarchaeota archaeon* isolate Bin-L-2 (light blue) superimposed with *Burkholderia* phage BEK TShP (salmon). (**d**) The model of the putative tail sheath protein found in the genome of *Erwinia* phage ENT90 (slate) superimposed with *Burkholderia* phage BEK TShP (salmon). (**e**) The model of the putative tail sheath protein found in the genome of *Flavobacterium* phage FPSV-S1 (green) superimposed with *Burkholderia* phage BEK TShP (salmon). (**f**) The model of the putative tail sheath protein found in the genome of *Ralstonia* phage RSY1 (blue) superimposed with *Burkholderia* phage BEK TShP (salmon). (**g**) The model of the putative sheath protein found in the genome of *Vibrio* phage vB_VpaM_MAR (yellow) superimposed with *Burkholderia* phage BEK TShP (salmon).

The smallest modelled bacteriophage sheath protein belongs to a representative of the *Tigrvirus* genus of the *Peduovirinae* subfamily of the *Myoviridae* family *Burkholderia* phage BEK. It consists of 341 aa and its spatial structure is very close to the structural common core (Figure 6a). Most of the protein has a structural similarity with the conserved core of experimentally determined structures found by alignment and shown in Figure 2c. The structural architecture of sheath proteins from bacteriocins, T6SS, and anti-feeding prophage can be described as one-domain structures.

This one-domain structure is shared by modelled *Peduovirinae* TShPs, representing seven genera of this subfamily. This type of structure is shared by a number of other bacteriophages and sheath proteins predicted in the genomes of bacteria and archaea (*Lokiarchaeota*, *Bathyarchaeota*, *Euryarchaeota*) (Figure 6, Table 2). The length of the modelled type 1 TShPs varies in the range 321–410 aa. The *Candidatus Bathyarchaeota archaeon* protein is structurally very similar to *Burkholderia* phage BEK (RMSD 1.3 Å), but the *Burkholderia* phage BEK TShP possesses an additional short N-terminal part of about 30 aa. The *Candidatus Bathyarchaeota archaeon* predicted sheath protein is the shortest modelled sequence with a length of 321 amino acid residues.

#### 3.4.2. Two-Domains Contractile Sheath Proteins (Type 2)

The remaining modelled contractile sheath proteins possess the part that is structurally similar to that of the one-domain contractile sheath proteins, which will be referred to as the “main domain”, but they also possess additional domains. For some modelled structures, it was not possible to determine clearly whether the part of the protein excluding the main domain can be counted as a single domain. It might be related to the complex composition of the remaining part or it was caused by the inaccuracy of modelling. The structural architecture of most of the remaining sheath proteins can, however, be described as consisting of two domains, one of which is the main conserved domain. As a rule, the additional domain included β-sheets-related motifs and often contained immunoglobulin-like (Ig-like) β-sandwiches and short α-helical parts.

**Figure 7:**
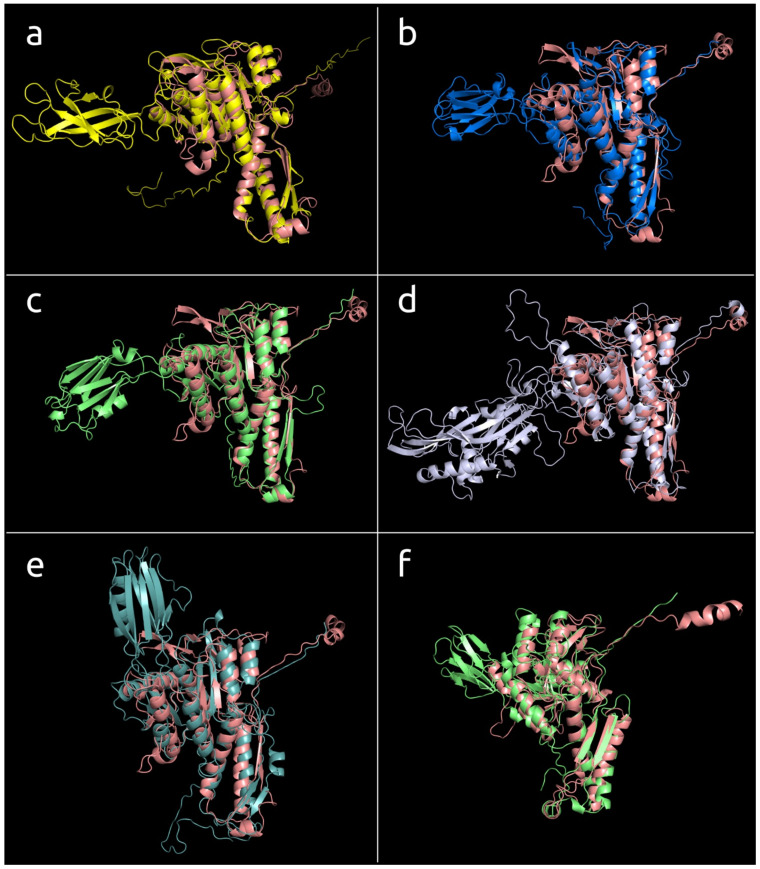

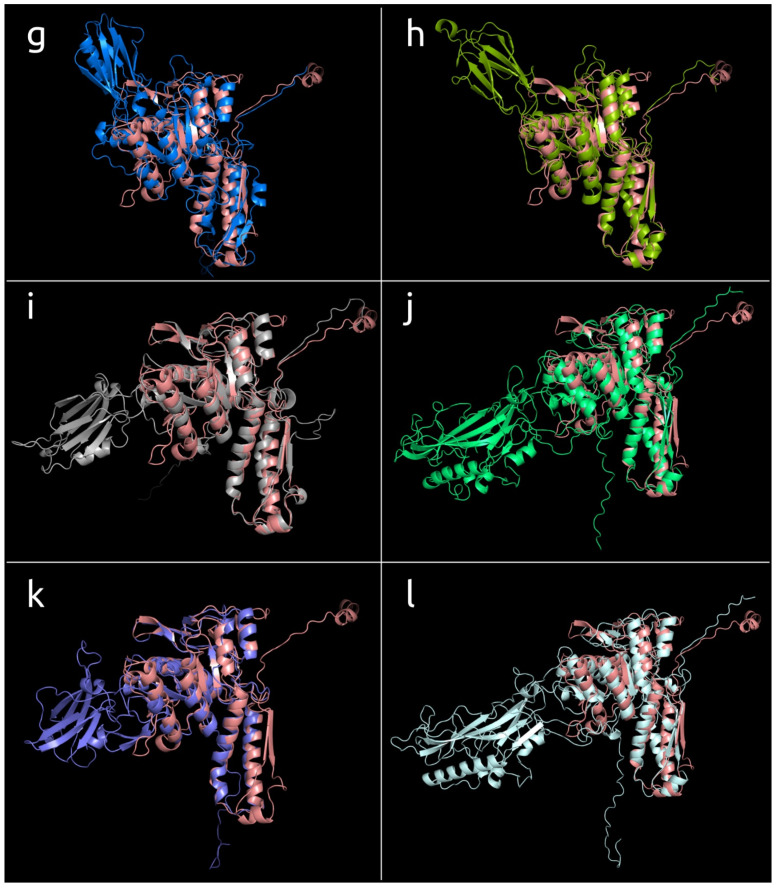
The models of the type 2 sheath proteins listed below superimposed with *Burkholderia* phage BEK TShP (painted salmon): (**a**) *Mycobacterium* phage Phabba, (**b**) *Brevibacillus* phage Jimmer2, (**c**) genome assembly attributed as *Thermoprotei archaeon* B19_G17, (**d**) *Erwinia* phage vB_EamM_RisingSun, (**e**) *Escherichia* phage Mu, (**f**) *Halobacterium* virus ChaoS9, (**g**) *Cellulophaga* phage phi38:2, (**h**) *Faecalibacterium* phage FP_Mushu, (**i**) *Gordonia* phage GMA6, (**j**) *Halocynthia* phage JM-2012, (**k**) genome assembly attributed as *Thermoplasmata archaeon* isolate B28_Guay1, (**l**) *Vibrio* phage BONAISHI.

All isolated archaeal viruses contained type 2 sheath proteins. Currently, isolated archaeal myoviruses are described as infecting *Halobacteria*. In addition, type 2 sheath proteins were found in phages assigned to the *Chaseviridae* family, different *Myoviridae* genera, *Paenibacillus* phage Lily, chromosomes and genome assemblies of Gram-positive and Gram-negative bacteria, and archaea attributed to phyla *Crenarchaeota*, *Euryarchaeota*, *Thaumarchaeota*, and *Thorarchaeota* (Figure 7, Supplementary File S1). The type 2 sheath proteins vary from 426 aa to 713 aa in size. The largest type 2 sheath proteins basically belong to Jumbo phages infecting gammaproteobacteria. The type 2 TShPs from isolated archaeal viruses were smaller than most other type 2 TShPs and contained an additional domain that was basically composed of β-sheets forming a β-sandwich.

#### 3.4.3. Multiple Domain Contractile Sheath Proteins (Type 3)

**Figure 8:**
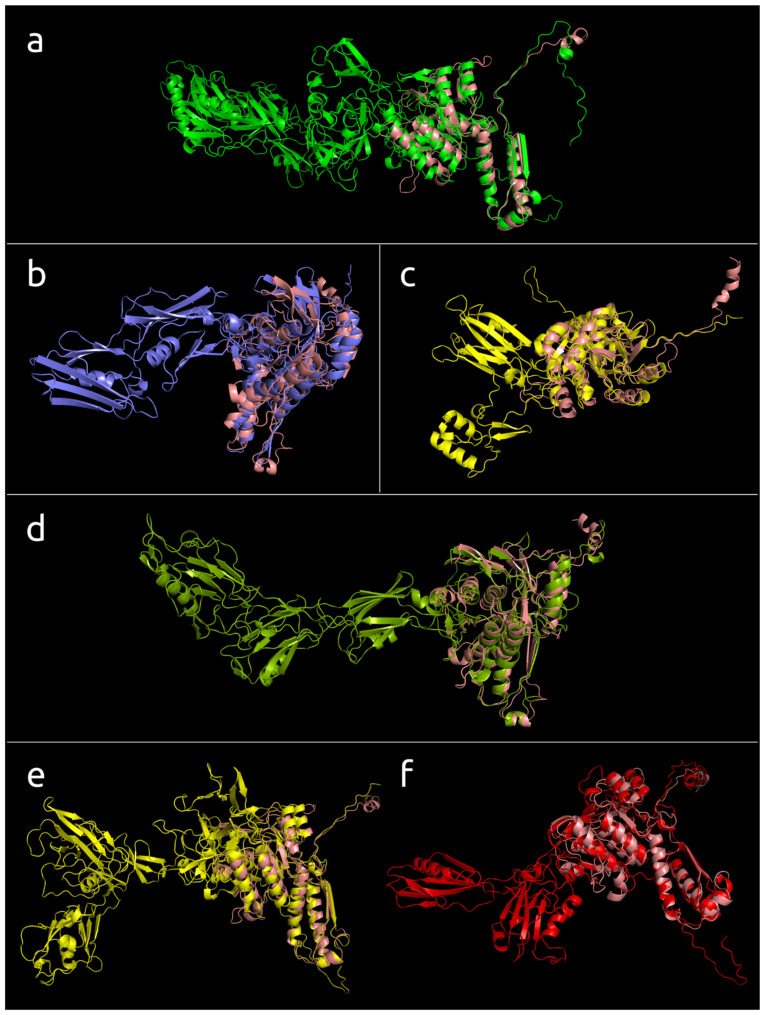

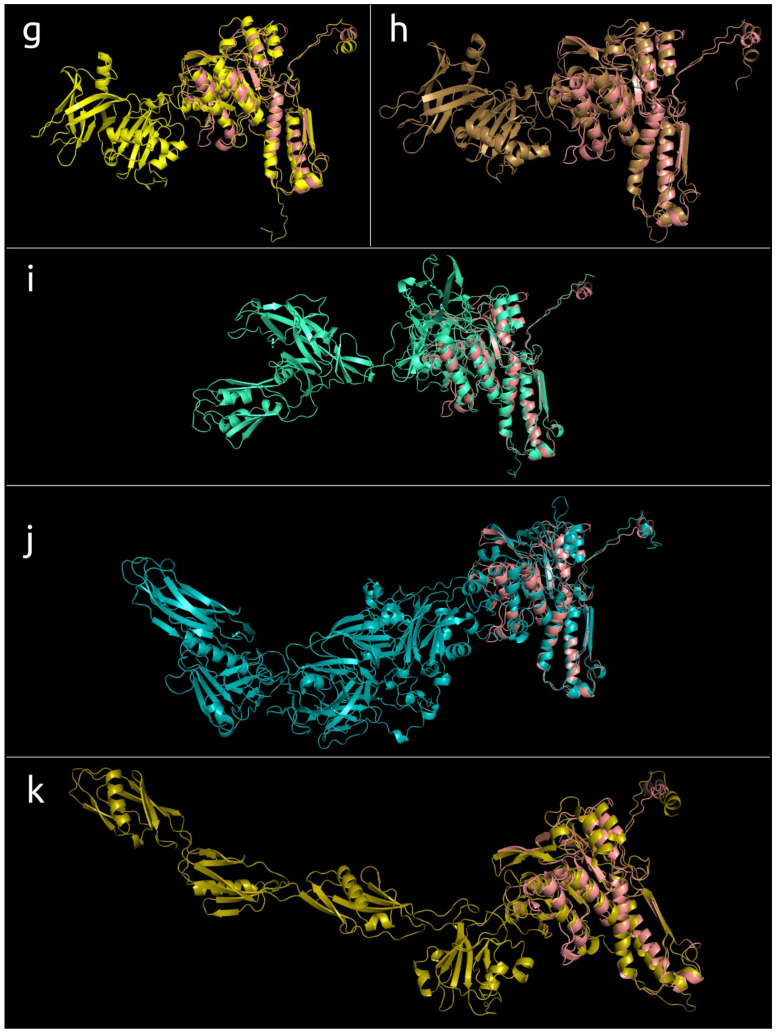
The models of the type 3 sheath proteins listed below superimposed with *Burkholderia* phage BEK TShP (painted salmon): (**a**) *Agrobacterium* phage Atu_ph07, (**b**) *Bacillus* phage BC01, (**c**) prophage TShP of *Halovivax ruber* XH-70, (**d**) genome assembly attributed as *Crenarchaeota archaeon* isolate__LB_CRA_1, (**e**) *Salicola* phage SCTP-2, (**f**) *Serratia* phage phiMAM1, (**g**) *Klebsiella* phage Miro, (**h**) *Kosakonia* phage Kc304, (**i**) *Klebsiella* phage vB_KleM_RaK2, (**j**) genome assembly attributed as phage Mad1_20_16, (**k**) *Ralstonia* phage RSP15.

**Figure 9:**
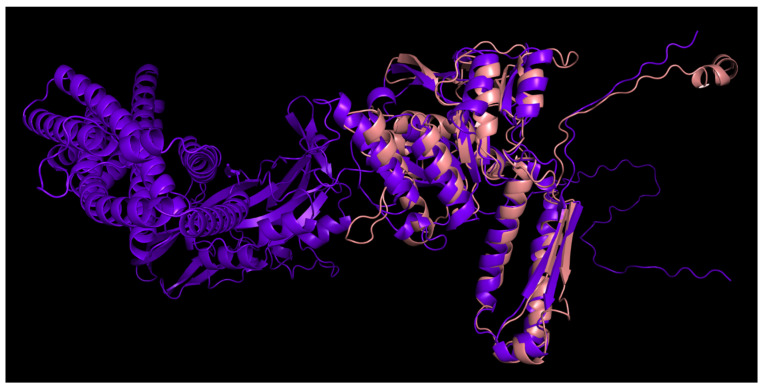
The 3D-model of the TShP of *Bacillus phages* PBS1 (violet) superimposed with the *Burkholderia* phage BEK TShP (salmon).

More than a third of the modelled structures showed a more complicated architecture than type 1 and type 2 proteins. The structural architecture of these proteins appears to be a further evolutionary development of type 2, and this architecture will be referred to as “type 3”. The modelled type 3 sheath proteins form a multi-domain structure composed of three and more domains (Figure 8). As in the case of type 2 sheath proteins, the additional domains often possessed an Ig-like β-sandwich structure sometimes accompanied by a few α-helices, but the sheath proteins from two related (ANI 99.0%) *Bacillus* phages, AR9 and PBS1, included additional domains comprised of mainly α-helices (Figure 9). As in the case of type 2 sheath proteins, as well as experimentally determined structures (Figure 4), the additional domains were located away from the part of the sheath protein that can contact the tail tube. Most phage genomes above 100 kbp in size encoded sheath proteins with three or more domains. The highest number of domains, five and more, were found in *Ackermannviridae* phages (genome size of approximately 140–170 kbp) and Jumbo phages (genome size of 200 kbp and bigger). Variants in the structural architecture of these proteins included additional domains formed by one region of the polypeptide chain, or two regions, one of which was closer to the N-terminus, and the other belonged to the returning part of the polypeptide chain located closer to the C-terminus.

### 3.5. Phylogenetic Analysis of Modelled Sheath Proteins

**Figure 10:**
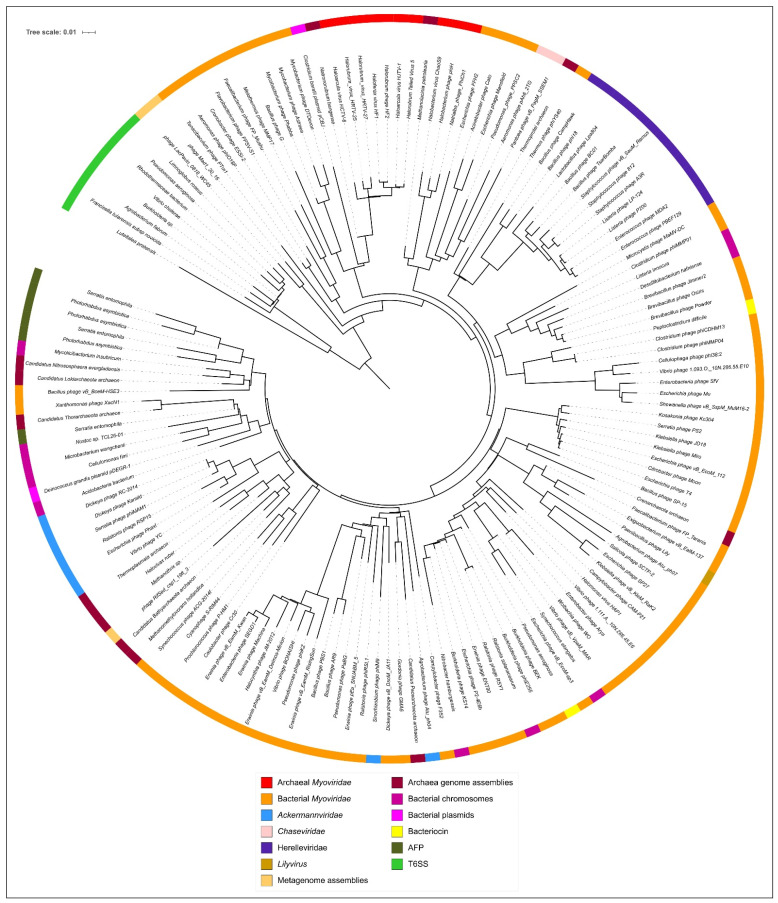
Circular tree constructed with 153 sheath proteins based on structural similarity assessed by mTM-align and clustered by BioNJ.

Multiple structural alignment with mTM-align algorithms [39,40] records pairwise TM-scores, a length-independent scoring function for measuring the similarity of two structures [67]. A matrix containing pairwise TM-scores was used for BioNJ clustering and for making inferences about a phylogenetic tree based on structural similarity (Figure 10). This tree differentiates the phage tail sheath proteins and other sheath proteins from type VI secretory system proteins and shows their slight similarity with the giant phage Mad1_20_16 and LacPavin_0818_WC45 TShPs. The archaeal sheath proteins and homologous sequences are placed in several different clades, but groups the haloarchaeal myoviruses belonging to genera *Haloferacalesvirus* and *Myohalovirus* in two monophyletic branches according to the taxonomy. Two haloarchaeal prophage TShPs were found to be structurally similar to the *Haloferacalesvirus* and *Myohalovirus* phage proteins. It is noteworthy that this tree places most of the archaeal sequences in the branches adjacent to Jumbo phages infecting Gram-positive bacteria.

Interestingly, this tree, based on structural similarity, indicates a closeness between the diffocin sheath protein from *Peptoclostridium difficile* and the tail sheath protein from *Clostridium* phage phiCDHM13 (genus *Sherbrookevirus* of the *Myoviridae* family). The proteins from the *Herelleviridae* and *Chaseviridae* families are placed in distinct clades, but two of the eight *Ackermannviridae* phages are in a separate branch and a different clade to the other six *Ackermannviridae* phages. Five of the six sheath proteins from anti-feeding prophages (AFP) are in a distinct clade adjacent to the clade containing *Bacillus* phage vB_BceM-HSE3 sheath proteins and homologous proteins found in archaeal and bacterial genomic sequences, but the remaining AFP sheath protein is in a different clade.

**Figure 11:**
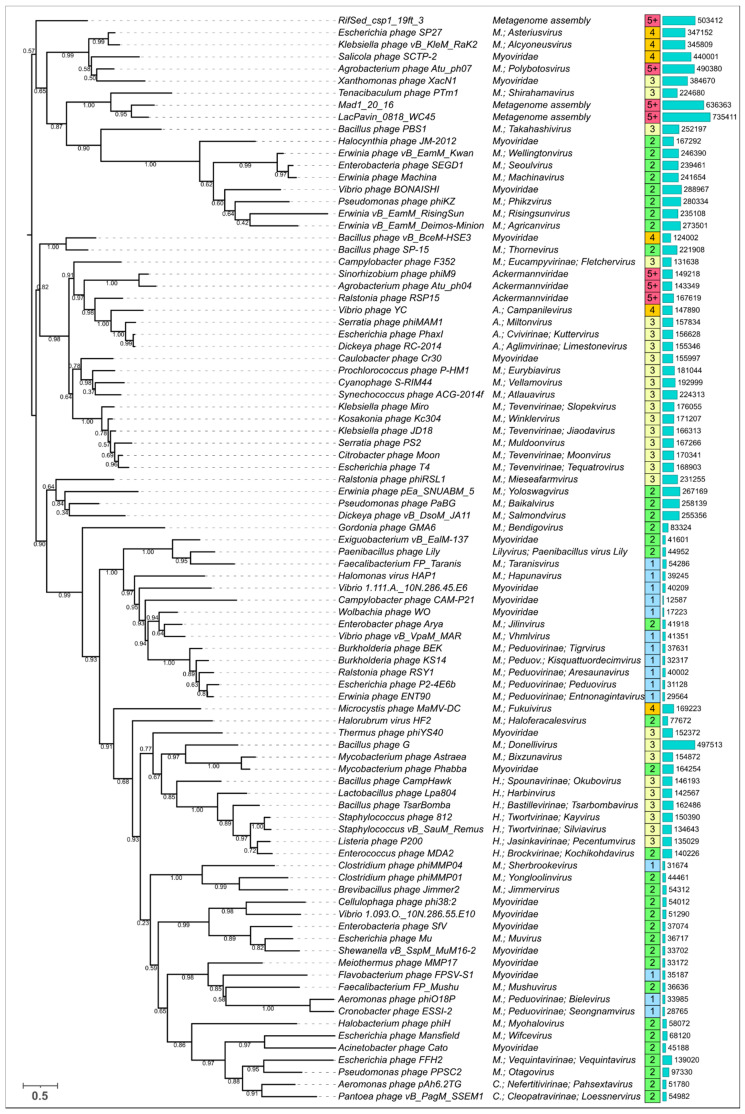
Best-scoring maximum likelihood (ML) phylogenetic tree constructed with 90 amino acid sequences for phage TShPs aligned with the mTM-align structural alignment algorithm and trimmed to the conserved “main” domain. NCBI taxonomy is shown to the right of the phage name. Total number of domains in the modelled structures are shown in the boxes to the right of the taxonomic assignment. The next column of bars indicate the phage genome length, as given in the NCBI phage GenBank database, and the numbers to the right correspond to the genome length. The genome of *Campylobacter* phage CAM-P21 seems to be incomplete, and the corresponding prophage sequences were used for some analyses. The numbers near the tree branches indicate the fraction of the bootstrap trees supporting the branch. The total number of bootstrap trees was 1000. The scale bar shows 0.5 estimated substitutions per site and the tree was rooted to the midpoint. The abbreviation “*M*” stands for *Myoviridae*.

A phylogenetic analysis was performed based on the alignment of amino acid sequences, which included only the conserved domain, and used statistical methods such as bootstrap analysis for estimating the robustness of the tree. A total of 90 trimmed amino acid sequences for TShPs were used for the tree shown in Figure 11, and the full amino acid sequences of these proteins are depicted in Supplementary Figure S1. This tree demonstrated greater consistency with the taxonomy. Interestingly, this tree also often placed Jumbo phages infecting Gram-positive bacteria and archaeal phages closer to the root of the branches that included phages infecting Gram-negative bacteria. This tree also put the representatives of *Haloferacalesvirus* and *Myohalovirus* genera into distinct clades in the same way as the tree based on overall structural similarity. Although this tree indicates the relatedness of structural architecture and taxonomy, this relatedness is not absolute. For example, the number of domains of all modelled *Peduovirinae* sheath proteins was constant and equal to one, but the number of domains of *Ackermannviridae* TShPs varied.

**Figure 12:**
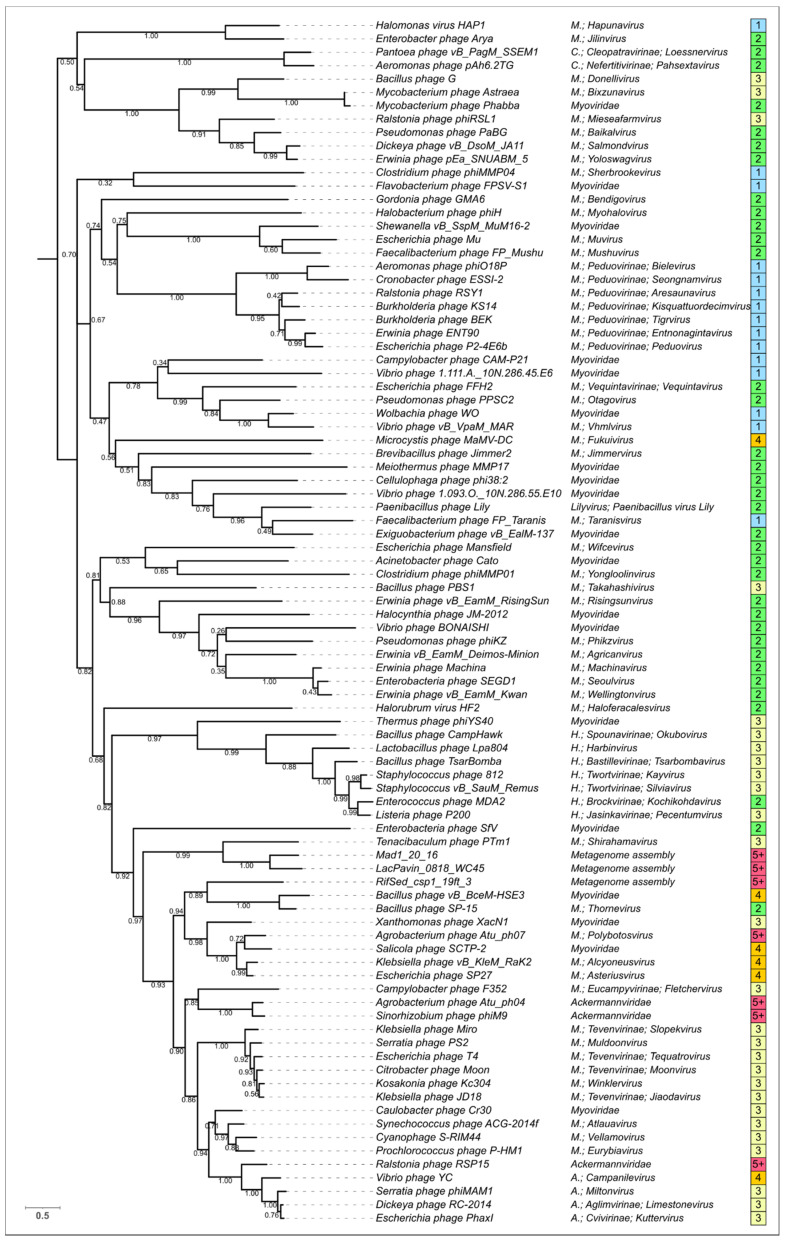
Best-scoring ML phylogenetic tree constructed with 90 amino acid sequences of phage major capsid protein aligned with MAFFT. The NCBI taxonomy is shown to the right of the phage name. Total number of domains in the modelled structures of the corresponding tail sheath proteins are shown in boxes to the right of the taxonomic assignment. The numbers near the tree branches indicate the fraction of the bootstrap trees supporting the branch. The total number of bootstrap trees was 1000. The scale bar shows 0.5 estimated substitutions per site and the tree was rooted to the midpoint. The abbreviation “*M*” stands for *Myoviridae*.

It is also interesting that the topology of a phylogenetic tree constructed using the alignment of primary amino acid sequences for major capsid proteins (Figure 12) shows a similar, but not identical, composition of the clades, and places archaeal *Haloferacalesvirus* and *Myohalovirus* viruses in distinct clades close to the phages infecting Gram-positive bacteria. The differences in topology might reflect both the problems with consistent phylogenetic analysis of highly divergent proteins and the consequences of the modular evolution of phages [68]. Phylogenies based on the large subunit of terminase (TerL) (Supplementary Figure S2) and tail tube protein (TTP) (Supplementary Figure S3) showed a similar situation with a partial closeness in the composition of clades and non-identical topology. The phylogenetic analysis of the tail tube protein had less bootstrap support than those for the TShP, MCP, and TerL phylogenies. This may be due to shorter sequences for the TTPs compared to the other listed proteins and the possibility of a comparatively high mutational rate.

## 4. Discussion

The analysis of experimentally determined and modelled structures indicates the presence of a common conserved core inherent for all analysed sheath proteins, including phage TShPs, type VI secretion system sheath proteins, bacteriocin, and anti-feeding prophage sheath proteins. It is also noteworthy that bacterial flagellin contains a multidomain structure with a conserved core composed of N- and C-termini of a polypeptide chain important for the flagellin self-assembly, which somewhat resembles TShPs [69,70].

Most of the phage TShPs studied in this report differed from non-phage sheath proteins due to the presence of additional domains. These domains seem to be located away from the part of the TShP that is in contact with the tail tube. Such a location would prevent perturbation of the tail assembly and the function of the contractile mechanism. In this way, the evolution of phage TShPs was conditioned by its biological role. Phylogenetic analysis on the basis of structural similarity indicated the relatedness of T6SS sheath proteins and the TShP of giant phages. This may indicate the ancient divergence of phage sheath proteins and T6SS. Further, the similarity between anti-feeding prophage sheath proteins, the TShPs of Gram-positive bacteria, and the predicted sheath proteins for archaea may also have an ancient origin and were a consequence of the specialisation of AFP. In contrast, diffocin and pyocin sheath proteins appear to arise later and can be polyphyletic. As a minimum, the *Peptoclostridium difficile* diffocin sheath protein is structurally closer to *Clostridium* phages than it is to all other sheath proteins, and the *Pseudomonas aeruginosa* pyocin sheath protein is structurally similar to the TShPs of phages infecting beta- and gammaproteobacteria. For the future, the origin of phage-like contractile machines will require dedicated research using a larger representative group.

The size of the phage tail sheath proteins and the number of additional domains correlated with the size of the genome, such that small phages possessed shorter one- or two-domain TShPs. This observation seems reasonable, since the additional domains are not essential for the assembly and operation of the contractile mechanism, but they will consume resources for carrying the extra genetic material and protein synthesis during the infection. Large phages often have multi-domain TShPs. The suggestion, here, is that the ancestral form of phage TShPs possessed one main domain, and that during the evolution of phages, accompanied by an increase in genome size, phage TShPs acquired additional domains. This process may have some common features with the acquisition of additional functional genes during Jumbo phage evolution [71].

The necessity of expending additional resources as a result of having larger sheath proteins must be justified by competitive advantages provided by the additional domains. Most additional domains in the studied TShPs exhibited an immunoglobulin-like fold. It might be hypothesised that the presence of additional Ig-like TShP domains assists the adhesion of phages to the bacteria. Ig-like domains have been shown to be the subject of common horizontal exchange between diverse classes of both lytic and temperate phages, and Ig-like domains “may play an accessory role in phage infection by weakly interacting with carbohydrates on the bacterial cell surface” [72]. A further hypothesis might be that these domains can participate in the formation of tail appendages detected for some large phages [73], since Ig-like domains often participate in protein-protein interactions [74], which, in turn, also promote cell adhesion. It might also be possible that the presence of additional domains facilitates an increase in the stability of the virion, which is vital for phages [75,76], by cementing the assembled tail exploiting the interactions between additional domains of TShPs. This proposal agrees with the suggestion of Nováček et al. based on the analysis of the cryo-EM reconstruction of *Staphylococcus* phage 812 [48] that an additional domain (named “domain II” in [48]) makes contact with domain III (which is part of conserved core, according to present research) “from a neighboring tail sheath protein probably stabilizing the tail sheath protein disk”. Interestingly, additional domains of phage T4 were supposed to be nonessential for tail sheath formation [1,77,78].

AlphaFold 2 software has shown an impressive level of accuracy in the modelling of proteins with experimentally determined tertiary structures. For 14 of the 15 structures, the RMSD was 0.59–1.33 Å. In one case (*Staphylococcus* phage 812), the RMSD was 3.27 Å for the contracted protein, and 2.83 Å for the native conformation. Low RMSD values could be a consequence of using templates, but comparative phylogenetic analysis using the structures of tail sheath proteins, sequences of major capsid proteins, and large subunits of terminase showed an identical or similar composition of clades, at least at the level of genera and subfamilies, supporting the results obtained from the AlphaFold 2 simulations.

It was previously shown that during contraction, the TShP subunits of phage T4 slide over each other with no apparent change in their structure [1], whereas the TShP of *Staphylococcus* phage 812 changes conformation during contraction [48]. For several analysed AlphaFold 2 models, the difference between the RMSD for experimentally determined structures in the contracted state and the models of the structures in an extended state was several-fold lower than the accuracy of the experimental methods. Therefore, it is hardly possible to draw conclusions if contracted or extended state is closer to the AlphaFold 2 model.

Phylogenetic analysis using the major capsid protein and terminase has traditionally been used to reveal taxonomic and evolutionary relationships between bacteriophages [63,79]. Now it seems that the structural and phylogenetic analysis of TShP could help in clarifying the evolutionary history of phages. Moreover, the results of AlphaFold 2 predictions could also be used together with other analytical methods for elucidating the evolutionary history of proteins and bacteriophages. Conversely, phylogenetic analyses that do not take into account structural features can be based on an erroneous evolutionary history, and incorrect alignments can lead to a flawed phylogeny, even though the statistical analysis (e.g., bootstrap values) might indicate high branch support, according to the principle of “garbage in–garbage out”. The incongruence of the topologies for different trees can be caused not only by the inaccuracy of structural predictions, but also by the independent evolution of different proteins as a consequence of modular evolution of bacteriophages [68]. Differences in the topology of phylogenetic trees can be observed for conserved proteins such as MCP and terminase, which was noted for some phages in previous research [80]. It is noteworthy that our phylogenetic analyses for tail sheath proteins and major capsid proteins resulted in the distant placement of archaeal viruses belonging to *Haloferacalesvirus* and *Myohalovirus* genera, yet they were closely related according to our terminase large subunit phylogeny.

The origin of archaeal phages is an exceptionally important question for evolutionary biology. During our search for homologs of sheath proteins in archaeal genomes, several putative sequences were found in the metagenomic assemblies of archaea, which had been classified as representatives of groups other than Haloarchaea. These findings could be the result of erroneous metagenomic binning, but a BLAST search also found homologs of myoviral major capsid proteins or terminase in dozens of draft genomes attributed as *Aenigmarchaeota*, Asgard group, *Bathyarchaeota*, *Korarchaeota*, *Nanoarchaeota*, *Pacearchaeota*, *Thaumarchaeota*, and *Woesearchaeota*, and homologs of myoviral terminase in the complete genomes of *Candidatus Caldarchaeum subterraneum* spp., *Candidatus Fermentimicrarchaeum limneticum* isolate Sv326, *Candidatus Heimdallarchaeota* archaeon spp., etc. It is known that the archaeal myoviruses isolated to date preferentially infect archaea from the *Euryarchaeota* phylum [81]. The probable presence of myoviral sequences in the genomes of other archaea could indicate a wider diversity for archaeal viruses than is currently expected. The presence of several domains in putative archaeal sheath proteins found in some presumably archaeal sequences (i.e., attributed as *Crenarchaeota archaeon* isolate LB_CRA_1 and *Candidatus Pacearchaeota archaeon* isolate ARS50) might suggest their prophage origin. The possible existence of myoviral prophages in non-euryarchaeal genomes needs very thorough analysis and verification. In addition, the results of our phylogenetic analysis of the structural similarity of sheath proteins suggests a polyphyletic origin for the predicted archaeal sheath proteins. If this assumption is correct, it is also possible that different groups of viruses with myoviral morphology existed before the divergence of the main archaeal and bacterial groups. Thus, the origin and early evolution of myoviruses requires dedicated evolutionary studies.

## 5. Conclusions

The results of our bioinformatic research on phage tail sheath proteins indicate the presence of a conserved core in all sheath proteins that is presumably responsible for tail assembly and the function of the myoviral contractile injection mechanism. The evolution of the phage tail sheath protein is accompanied by the incorporation of additional domains, many of which contain an immunoglobulin-like β-sandwiches fold. The functional requirements of the phage contractile injection system has resulted in the appearance of a specific structural architecture for the phage tail sheath proteins that includes the presence of a conserved domain, composed of both N-terminal and C-terminal parts in contact with the phage tail tube, and additional domains, which could facilitate adhesion to the host cell.

## Figures and Tables

**Table 1:** List of experimentally determined structures for sheath proteins and related structures.

PDB Code	Description	Organism	Resolution	Method	References
3FO8	Crystal structure of the bacteriophage T4 tail sheath protein, protease-resistant fragment gp18PR	*Escherichia* phage T4	1.8 Å	X-ray diffraction	[1]
3FOA	Crystal structure of the bacteriophage T4 tail sheath protein, deletion mutant gp18M	*Escherichia* phage T4	3.5 Å	X-ray diffraction	[1]
3HXL	Crystal structure of the sheath tail protein (DSY3957) from *Desulfitobacterium hafniense*	*Desulfitobacterium hafniense*	1.90 Å	X-ray diffraction	[46]
3LML	Crystal structure of the sheath tail protein Lin1278 from *Listeria innocua*, Northeast Structural Genomics Consortium Target LkR115	*Listeria innocua*	3.3 Å	X-ray diffraction	[47]
3SPE	Crystal structure of the tail sheath protein protease-resistant fragment from bacteriophage phiKZ	*Pseudomonas* phage phiKZ	2.4 Å	X-ray diffraction	[2]
5LI4	Bacteriophage phi812K1-420 (*Staphylococcus* phage 812) tail sheath protein after contraction. This structure is related to 5LI2, 5LII, 5LIJ	*Staphylococcus* phage 812	4.2 Å	Electron microscopy	[48]
6GKW	Crystal structure of the R-type bacteriocin (diffocin) sheath protein CD1363 from *Clostridium difficile* 630 in the pre-assembled state	*Clostridium difficile*	1.9 Å	X-ray diffraction	[49]
6PYT	CryoEM structure of precontracted pyocin R2 trunk from *Pseudomonas aeruginosa*	*Pseudomonas aeruginosa*	2.9 Å	Electron microscopy	[50]
3J9O	CryoEM structure of a type VI secretion system from *Francisella tularensis* subsp. *novicida* U112	*Francisella tularensis* subsp. *novicida*	3.70 Å	Electron microscopy	[51]
5N8N	CryoEM structure of contracted sheath of a *Pseudomonas aeruginosa* type VI secretion system consisting of TssB1 and TssC	*Pseudomonas aeruginosa*	3.28 Å	Electron microscopy	[52]
3J9G	Atomic model of the VipA/VipB, the type VI secretion system contractile sheath of *Vibrio cholerae*	*Vibrio cholerae*	3.5 Å	Electron microscopy	[53]
6RAO	Cryo-EM structure of the anti-feeding prophage (AFP) baseplate for *Serratia entomophila.* This structure is related to 6RAP, 6RBK, 6RBN, 6RC8, 6RGL	*Serratia entomophila*	3.1 Å	Electron microscopy	[4]
6J0B	Cryo-EM structure of an extracellular contractile injection system (CIS), PVC sheath-tube complex in extended state from *Photorhabdus asymbiotica* subsp. *asymbiotica* ATCC 43949	*Photorhabdus asymbiotica* subsp. *asymbiotica* ATCC 43949	2.9 Å	Electron microscopy	[3]
7AE0	Cryo-EM structure of an extracellular contractile injection system from the marine bacterium *Algoriphagus machipongonensis* with the sheath-tube module in its extended state. This structure is related to 7ADZ, 7AE0, 7AEB, 7AEF, 7AEK	*Algoriphagus machipongonensis*	2.4 Å	Electron microscopy	[54]
7B5I	Cryo-EM structure of the contractile injection system cap complex from *Anabaena* PCC7120	*Nostoc* sp.	2.8 Å	Electron microscopy	[55]

**Table 2:** List of 153 contractile sheath proteins and homologous sequences for which the tertiary structures have been modelled.

#	Organism Name (AFP, Anti-Feeding Prophage; BCN, Bacteriocin; CHR, Chromosome or Genome Assembly; PMD, Plasmid; T6SS, Type VI Secretion System)	NCBI Taxonomy	Length of Sheath Protein, Amino Acid Residues	Number of Domains in the Modelled Structure
1	*Acinetobacter phage Cato*	*Myoviridae*	487	2
2	*Aeromonas phage pAh6_2TG*	*Chaseviridae*; *Nefertitivirinae*; *Pahsextavirus*	472	2
3	*Aeromonas phage phiO18P*	*Myoviridae*; *Peduovirinae*; *Bielevirus*	370	1
4	*AFP-6J0B-SP1 Photorhabdus asymbiotica ATCC43949*	*Gammaproteobacteria*; *Enterobacterales*; *Morganellaceae*	355	1
5	*AFP-6J0N-SP2 Photorhabdus asymbiotica ATCC43949*	*Gammaproteobacteria*; *Enterobacterales*; *Morganellaceae*	440	1
6	*AFP-6J0N-SP3 Photorhabdus asymbiotica ATCC43949*	*Gammaproteobacteria*; *Enterobacterales*; *Morganellaceae*	424	1
7	*AFP-6RAO-SP3 Serratia entomophila*	*Gammaproteobacteria*; *Enterobacterales*; *Yersiniaceae*	417	1
8	*AFP-6RBN-SP1 Serratia entomophila*	*Gammaproteobacteria*; *Enterobacterales*; *Yersiniaceae*; *Serratia*	354	1
9	*AFP-6RBN-SP2 Serratia entomophila*	*Gammaproteobacteria*; *Enterobacterales*; *Yersiniaceae*; *Serratia*	451	1
10	*Agrobacterium phage Atu_ph04*	*Ackermannviridae*	838	5+
11	*Agrobacterium phage Atu_ph07*	*Myoviridae*; *Polybotosvirus*	1086	5+
12	*Bacillus phage AR9*	*Myoviridae*	987	3
13	*Bacillus phage BC01*	*Herelleviridae*; *Bastillevirinae*; *Tsarbombavirus*	568	3
14	*Bacillus phage CampHawk*	*Herelleviridae*; *Spounavirinae*; *Okubovirus*	571	3
15	*Bacillus phage G*	*Myoviridae*; *Donellivirus*	579	3
16	*Bacillus phage PBS1*	*Myoviridae*; *Takahashivirus*	987	3
17	*Bacillus phage phi18*	*Herelleviridae*; *Spounavirinae*; *Okubovirus*	571	3
18	*Bacillus phage SP-15*	*Myoviridae*; *Thornevirus*	494	2
19	*Bacillus phage TsarBomba*	*Herelleviridae*; *Bastillevirinae*; *Tsarbombavirus*	568	3
20	*Bacillus phage vB_BceM-HSE3*	*Myoviridae*	727	4
21	*BCN-6GKW-Peptoclostridium difficile*	*Firmicutes*; *Clostridia*; *Clostridiales*; *Peptostreptococcaceae*; *Clostridioides*	356	1
22	*BCN-6PYT-Pseudomonas aeruginosa PAO1*	*Gammaproteobacteria*; *Pseudomonadales*; *Pseudomonadaceae*; *Pseudomonas*	386	1
23	*Brevibacillus phage Jimmer2*	*Myoviridae*; *Jimmervirus*	437	2
24	*Brevibacillus phage Osiris*	*Myoviridae*; *Jimmervirus*	437	2
25	*Brevibacillus phage Powder*	*Myoviridae*; *Jimmervirus*	437	2
26	*Burkholderia phage BEK*	*Myoviridae*; *Peduovirinae*; *Tigrvirus*	342	1
27	*Burkholderia phage KS14*	*Myoviridae*; *Peduovirinae*; *Kisquattuordecimvirus*	391	1
28	*Burkholderia phage phiE255*	*Myoviridae*; *Bcepmuvirus*	477	2
29	*Campylobacter phage CAM-P21*	*Myoviridae*	397	1
30	*Campylobacter phage F352*	*Myoviridae*; *Eucampyvirinae*; *Fletchervirus*	636	3
31	*Caulobacter phage Cr30*	*Myoviridae*	688	3
32	*Cellulophaga phage phi38:2*	*Myoviridae*	508	2
33	*CHR-3HXL-Desulfitobacterium hafniense*	*Firmicutes*; *Clostridia*; *Clostridiales*; *Peptococcaceae*; *Desulfitobacterium*	446	2
34	*CHR-3LML-Listeria innocua*	*Firmicutes*; *Bacilli*; *Bacillales*; *Listeriaceae*; *Listeria*	460	2
35	*CHR-Acidobacteria bacterium Mor1*	*Acidobacteria*	410	1
36	*CHR-Candidatus Bathyarchaeota archaeon isolate Bin-L-2*	*Candidatus Bathyarchaeota*	321	1
37	*CHR-Candidatus Lokiarchaeota archaeon isolate TEKIR_9*	*Asgard group*; *Candidatus Lokiarchaeota*	369	1
38	*CHR-Candidatus Nitrososphaera evergladensis SR1*	*Thaumarchaeota*; *Nitrososphaeria*; *Nitrososphaerales*; *Nitrososphaeraceae*	521	2
39	*CHR-Candidatus Pacearchaeota archaeon isolate ARS50*	*Candidatus Pacearchaeota*	634	3
40	*CHR-Candidatus Thorarchaeota archaeon isolate 2_13*	*Asgard group*; *Candidatus Thorarchaeota*	577	2
41	*CHR-Cellulomonas fimi ATCC 484*	*Actinobacteria*; *Micrococcales*; *Cellulomonadaceae*	523	2
42	*CHR-Crenarchaeota archaeon isolate LB_CRA_1*	*Crenarchaeota*	805	4
43	*CHR-Halovivax ruber XH-70*	*Euryarchaeota*; *Stenosarchaea group*; *HaloNatrialbales*; *Natrialbaceae*	574	3
44	*CHR-Methanolacinia_petrolearia_DSM_11571*	*Euryarchaeota*; *Methanomicrobia*; *Methanomicrobiales*; *Methanomicrobiaceae*; *Methanolacinia*	343	1
45	*CHR-Methanomethylovorans hollandica DSM 15978*	*Euryarchaeota*; *Stenosarchaea group*; *Methanomicrobia*; *Methanosarcinales*; *Methanosarcinaceae*	540	2
46	*CHR-Methanothrix* sp. *isolate bin.308 Contig_420493*	*Euryarchaeota*; *Stenosarchaea group*; *Methanomicrobia*; *Methanosarcinales*; *Methanosaetaceae*	509	2
47	*CHR-Microbacterium wangchenii strain dk512*	*Actinobacteria*; *Micrococcales*; *Microbacteriaceae*	520	2
48	*CHR-Mycolicibacterium insubricum JCM 16366*	*Actinobacteria*; *Corynebacteriales*; *Mycobacteriaceae*	508	2
49	*CHR-Natronorubrum bangense strain JCM 10635*	*Euryarchaeota*; *Stenosarchaea group*; *HaloNatrialbales*; *Natrialbaceae*	348	1
50	*CHR-Nitrobacter hamburgensis X14*	*Alphaproteobacteria*; *Rhizobiales*; *Bradyrhizobiaceae*	478	2
51	*CHR-Nostoc* sp. *TCL26-01*	*Cyanobacteria*; *Nostocales*; *Nostocaceae*; *Nostoc*	474	2
52	*CHR-Ralstonia solanacearum strain UW774*	*Betaproteobacteria*; *Burkholderiales*; *Burkholderiaceae*	476	2
53	*CHR-Synechococcus elongatus PCC 6301*	*Cyanobacteria*; *Synechococcales*; *Synechococcaceae*	474	2
54	*CHR-Thermoplasmata archaeon isolate B28_G1*	*Euryarchaeota*; *Diaforarchaea group*; *Thermoplasmata*	436	2
55	*CHR-Thermoprotei archaeon B19_G17*	*Archaea*; *Crenarchaeota*; *Thermoprotei*	452	2
56	*Citrobacter phage Moon*	*Myoviridae*; *Tevenvirinae*; *Moonvirus*	658	3
57	*Clostridium phage phiCDHM13*	*Myoviridae*; *Sherbrookevirus*	355	1
58	*Clostridium phage phiMMP01*	*Myoviridae*; *Yongloolinvirus*	436	2
59	*Clostridium phage phiMMP04*	*Myoviridae*; *Sherbrookevirus*	355	1
60	*Cronobacter phage ESSI-2*	*Myoviridae*; *Peduovirinae*; *Seongnamvirus*	375	1
61	*Cyanophage S-RIM44*	*Myoviridae*; *Vellamovirus*	635	3
62	*Dickeya phage Kamild*	*Ackermannviridae*; *Aglimvirinae*; *Limestonevirus*	632	3
63	*Dickeya phage RC-2014*	*Ackermannviridae*; *Aglimvirinae*; *Limestonevirus*	632	3
64	*Dickeya phage vB_DsoM_JA11*	*Myoviridae*; *Salmondvirus*	558	2
65	*Enterobacter phage Arya*	*Myoviridae*; *Jilinvirus*	477	2
66	*Enterobacteria phage SEGD1*	*Myoviridae*; *Seoulvirus*	681	2
67	*Enterobacteria phage SfV*	*Myoviridae*	498	2
68	*Enterococcus phage MDA2*	*Herelleviridae*; *Brockvirinae*; *Kochikohdavirus*	569	2
69	*Enterococcus phage PBEF129*	*Herelleviridae*; *Brockvirinae*; *Kochikohdavirus*	569	3
70	*Erwinia phage ENT90*	*Myoviridae*; *Peduovirinae*; *Entnonagintavirus*	389	1
71	*Erwinia phage Machina*	*Myoviridae*; *Machinavirus*	680	2
72	*Erwinia phage pEa_SNUABM_5*	*Myoviridae*; *Yoloswagvirus*	563	2
73	*Erwinia phage vB_EamM_Deimos-Minion*	*Myoviridae*; *Agricanvirus*	695	2
74	*Erwinia phage vB_EamM_Kwan*	*Myoviridae*; *Wellingtonvirus*	681	2
75	*Erwinia phage vB_EamM_RisingSun*	*Myoviridae*; *Risingsunvirus*	713	2
76	*Escherichia phage FFH2*	*Myoviridae*; *Vequintavirinae*	458	2
77	*Escherichia phage Mansfield*	*Myoviridae*; *Wifcevirus*	512	2
78	*Escherichia phage Mu*	*Myoviridae*; *Muvirus*	495	2
79	*Escherichia phage P2-4E6b*	*Myoviridae*; *Peduovirinae*; *Peduovirus*	396	1
80	*Escherichia phage PhaxI*	*Ackermannviridae*; *Cvivirinae*; *Kuttervirus*	631	3
81	*Escherichia phage SP27*	*Myoviridae*; *Asteriusvirus*	887	4
82	*Escherichia phage T4*	*Myoviridae*; *Tevenvirinae*; *Tequatrovirus*	659	3
83	*Escherichia phage vB_EcoM_112*	*Myoviridae*; *Tevenvirinae*; *Tequatrovirus*	659	3
84	*Escherichia phage vB_EcoM-ep3*	*Myoviridae*; *Jilinvirus*	475	2
85	*Exiguobacterium phage vB_EalM-137*	*Myoviridae*	482	2
86	*Faecalibacterium phage FP_Mushu*	*Myoviridae*; *Mushuvirus*	481	2
87	*Faecalibacterium phage FP_Taranis*	*Myoviridae*; *Taranisvirus*	384	1
88	*Flavobacterium phage FPSV-S1*	*Myoviridae*	390	1
89	*Gordonia phage GMA6*	*Myoviridae*; *Bendigovirus*	482	2
90	*Haloarcula virus HCTV-6*	*Myoviridae*; *Haloferacalesvirus*	437	2
91	*Haloarcula virus HJTV-1*	*Myoviridae*; *Haloferacalesvirus*	430	2
92	*Halobacterium phage phiH*	*Myoviridae*; *Myohalovirus*	432	2
93	*Halobacterium virus ChaoS9*	*Myoviridae*; *Myohalovirus*	434	2
94	*Halocynthia phage JM-2012*	*Myoviridae*	681	2
95	*Haloferax virus HF1*	*Myoviridae*; *Haloferacalesvirus*	430	2
96	*Halomonas virus HAP1*	*Myoviridae*; *Hapunavirus*	388	1
97	*Halorubrum phage HF2*	*Myoviridae*; *Haloferacalesvirus*	430	2
98	*Halorubrum Tailed Virus 5*	*Myoviridae*; *Haloferacalesvirus*	430	2
99	*Halorubrum_virus_HRTV-25*	*Myoviridae*; *Haloferacalesvirus*	431	2
100	*Halorubrum_virus_HRTV-27*	*Myoviridae*; *Haloferacalesvirus*	430	2
101	*Klebsiella phage JD18*	*Myoviridae*; *Tevenvirinae*; *Jiaodavirus*	657	3
102	*Klebsiella phage Miro*	*Myoviridae*; *Tevenvirinae*; *Slopekvirus*	663	3
103	*Klebsiella phage vB_KleM_RaK2*	*Myoviridae*; *Alcyoneusvirus*	888	4
104	*Kosakonia phage Kc304*	*Myoviridae*; *Winklervirus*	660	3
105	*Lactobacillus phage Lpa804*	*Herelleviridae*; *Harbinvirus*	612	3
106	*Listeria phage LP-124*	*Herelleviridae*; *Jasinkavirinae*; *Pecentumvirus*	562	3
107	*Listeria phage P200*	*Herelleviridae*; *Jasinkavirinae*; *Pecentumvirus*	562	3
108	*Meiothermus phage MMP17*	*Myoviridae*	472	2
109	*Microcystis phage MaMV-DC*	*Myoviridae*; *Fukuivirus*	774	4
110	*Mycobacterium phage Astraea*	*Myoviridae*; *Bixzunavirus*	581	3
111	*Mycobacterium phage DTDevon*	*Myoviridae*; *Bixzunavirus*	581	3
112	*Mycobacterium phage Phabba*	*Myoviridae*	482	2
113	*Natrialba_phage_PhiCh1*	*Myoviridae*; *Myohalovirus*	426	2
114	*Paenibacillus phage Lily*	*Lilyvirus*; *Paenibacillus virus Lily*	478	2
115	*Pantoea phage vB_PagM_SSEM1*	*Chaseviridae*; *Cleopatravirinae*; *Loessnervirus*	483	2
116	*phage LacPavin_0818_WC45*	*metagenome assembly*	1283	5+
117	*phage Mad1_20_16*	*metagenome assembly*	1248	5+
118	*phage RifSed_csp1_19ft_3*	*metagenome assembly*	881	5+
119	*PMD-Clostridium baratii str Sullivan plasmid pCBJ*	*Firmicutes*; *Clostridia*; *Clostridiales*; *Clostridiaceae*	814	4
120	*PMD-Deinococcus grandis ATCC 43672 plasmid pDEGR-1*	*Deinococcus-Thermus*; *Deinococci*; *Deinococcales*; *Deinococcaceae*	539	2
121	*Prochlorococcus phage P-HM1*	*Myoviridae*; *Eurybiavirus*	669	3
122	*Pseudomonas phage PaBG*	*Myoviridae*; *Baikalvirus*	547	2
123	*Pseudomonas phage phiKZ*	*Myoviridae*; *Phikzvirus*	695	2
124	*Pseudomonas_phage_PPSC2*	*Myoviridae*; *Otagovirus*	427	2
125	*Ralstonia phage phiRSL1*	*Myoviridae*; *Mieseafarmvirus*	648	3
126	*Ralstonia phage RSP15*	*Ackermannviridae*	826	5+
127	*Ralstonia phage RSY1*	*Myoviridae*; *Peduovirinae*; *Aresaunavirus*	391	1
128	*Salicola phage SCTP-2*	*Myoviridae*	955	4
129	*Serratia phage phiMAM1*	*Ackermannviridae*; *Miltonvirus*	636	3
130	*Serratia phage PS2*	*Myoviridae*; *Muldoonvirus*	663	3
131	*Shewanella phage vB_SspM_MuM16-2*	*Myoviridae*	493	2
132	*Sinorhizobium phage phiM9*	*Ackermannviridae*	838	5+
133	*Staphylococcus phage 812*	*Herelleviridae*; *Twortvirinae*; *Kayvirus*	587	3
134	*Staphylococcus phage A3R*	*Herelleviridae*; *Twortvirinae*; *Kayvirus*	587	3
135	*Staphylococcus phage vB_SauM_Remus*	*Herelleviridae*; *Twortvirinae*; *Silviavirus*	586	3
136	*Synechococcus phage ACG-2014f*	*Myoviridae*; *Atlauavirus*	731	3
137	*T6SS-3J9G-Vibrio cholerae*	*Gammaproteobacteria*; *Vibrionales*; *Vibrionaceae*	432	1
138	*T6SS-3J9O-Francisella tularensis subsp novicida*	*Gammaproteobacteria*; *Thiotrichales*; *Francisellaceae*	506	1
139	*T6SS-5N8N-Pseudomonas aeruginosa*	*Gammaproteobacteria*; *Pseudomonadales*; *Pseudomonadaceae*	498	1
140	*T6SS-Agrobacterium fabrum C58*	*Alphaproteobacteria*; *Hyphomicrobiales*; *Rhizobiaceae*	493	1
141	*T6SS-Burkholderia sp MSMB0852*	*Betaproteobacteria*; *Burkholderiales*; *Burkholderiaceae*; *Burkholderia*	499	1
142	*T6SS-Limnoglobus roseus strain PX52*	*Planctomycetes*; *Planctomycetia*; *Gemmatales*; *Gemmataceae*	491	1
143	*T6SS-Luteitalea pratensis DSM 100886*	*Acidobacteria*; *Vicinamibacteria*; *Vicinamibacteraceae*	493	1
144	*T6SS-Rhodothermaceae bacterium RA*	*Bacteroidetes*; *Bacteroidetes Order II. Incertae sedis*; *Rhodothermaceae*	509	1
145	*Tenacibaculum phage PTm1*	*Myoviridae*; *Shirahamavirus*	1032	3
146	*Thermus phage phiYS40*	*Myoviridae*	648	3
147	*Vibrio phage 1.093.O._10N.286.55.E10*	*Myoviridae*	486	2
148	*Vibrio phage 1.111.A._10N.286.45.E6*	*Myoviridae*	378	1
149	*Vibrio phage BONAISHI*	*Myoviridae*	682	2
150	*Vibrio phage vB_VpaM_MAR*	*Myoviridae*; *Vhmlvirus*	386	1
151	*Vibrio phage YC*	*Ackermannviridae*; *Campanilevirus*	756	4
152	*Wolbachia phage WO*	*Myoviridae*	383	1
153	*Xanthomonas phage XacN1*	*Myoviridae*	714	3

## Supplementary Materials

The following are available online at https://www.mdpi.com/article/10.3390/v14061148/s1. Figure S1. Best-scoring maximum likelihood phylogenetic tree constructed with 90 amino acid sequences of phage TShPs aligned with the mTM-align structural alignment algorithm. The NCBI taxonomy is shown to the right of the phage name. The numbers near the tree branches indicate the fraction of the bootstrap trees supporting the branch. The total number of bootstrap trees was 1000. The scale bar shows 0.5 estimated substitutions per site and the tree was rooted to the midpoint. Figure S2. Best-scoring ML phylogenetic tree constructed with 90 amino acid sequences of phage terminase large subunit aligned with MAFFT. The NCBI taxonomy is shown to the right of the phage name. Total number of domains in the modelled structures of the corresponding tail sheath proteins are shown in the column to the right of the taxonomic assignment. The numbers near the tree branches indicate the fraction of the bootstrap trees supporting the branch. The total number of bootstrap trees was 1000. The scale bar shows 0.5 estimated substitutions per site and the tree was rooted to the midpoint. Figure S3. Best-scoring ML phylogenetic tree constructed with 90 amino acid sequences of phage tail tube protein aligned with MAFFT. The NCBI taxonomy is shown to the right of the phage name. Total number of domains in the modelled structures of the corresponding tail sheath proteins are shown in the column to the right of the taxonomic assignment. The numbers near the tree branches indicate the fraction of the bootstrap trees supporting the branch. The total number of bootstrap trees was 1000. The scale bar shows 0.5 estimated substitutions per site and the tree was rooted to the midpoint. File S1. Fasta sequences of the modelled proteins. File S2. Best-ranked PDB structures modelled with AlphaFold 2.
