# Supplementary figures and images for: Evolution of Phage Tail Sheath Protein
Source: Viruses. 2022 May 26;14(6):1148. doi: 10.3390/v14061148 (PMC9230969; doi:10.3390/v14061148)

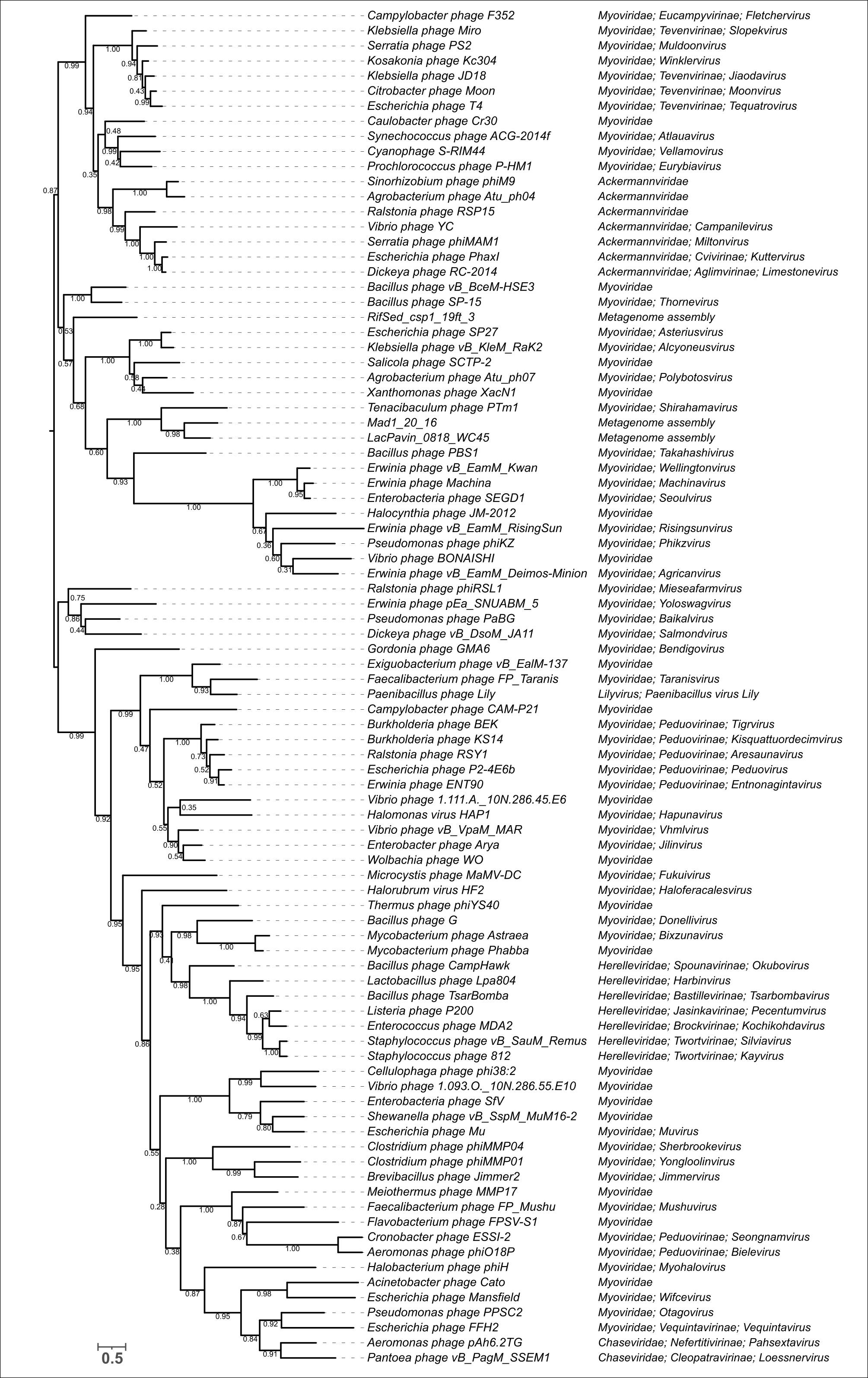

Supplement: Supplementary file 1 [file viruses-14-01148-s001.zip › Supplementary_Figure_S1.jpg]

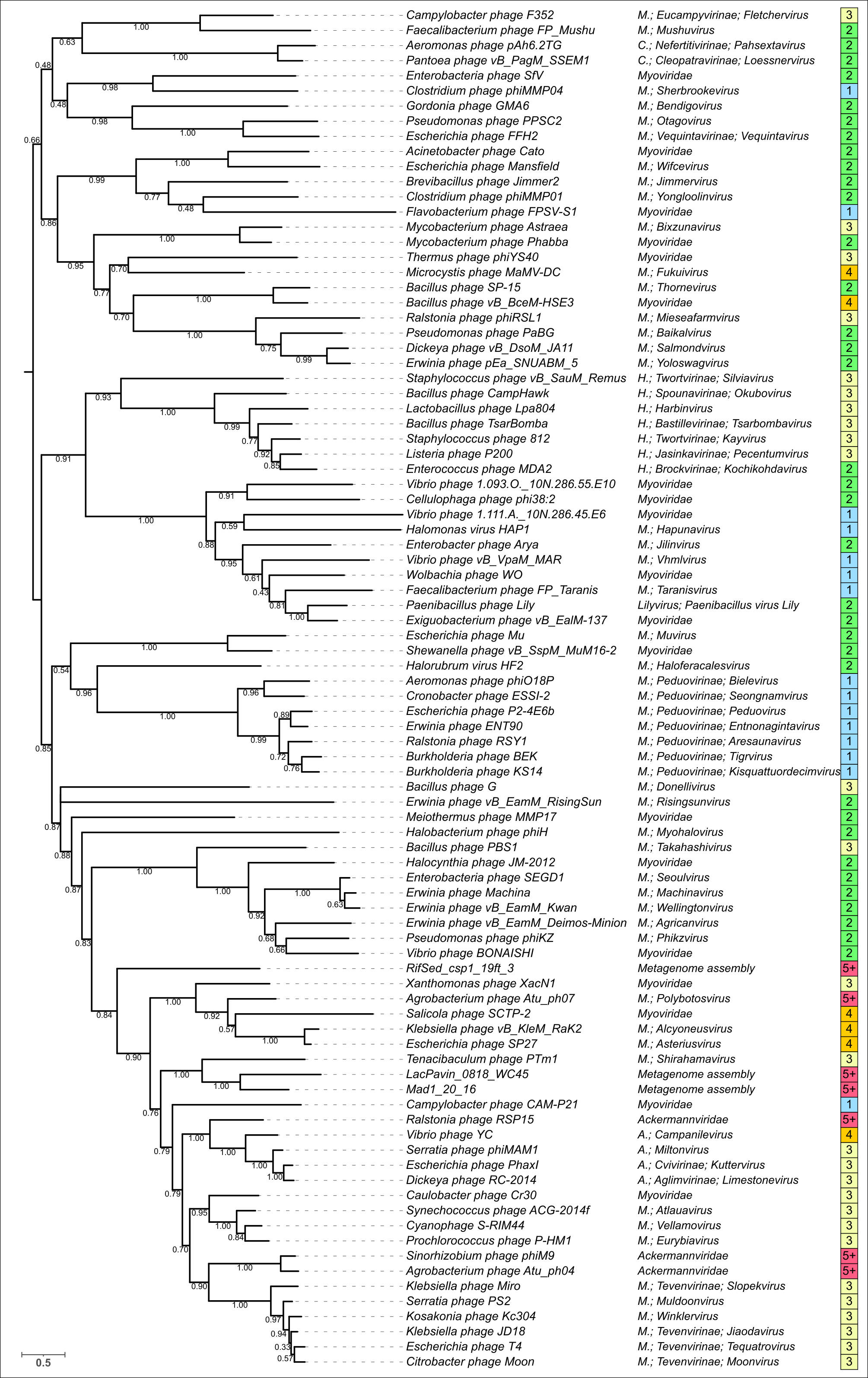

Supplement: Supplementary file 1 [file viruses-14-01148-s001.zip › Supplementary_Figure_S2.jpg]

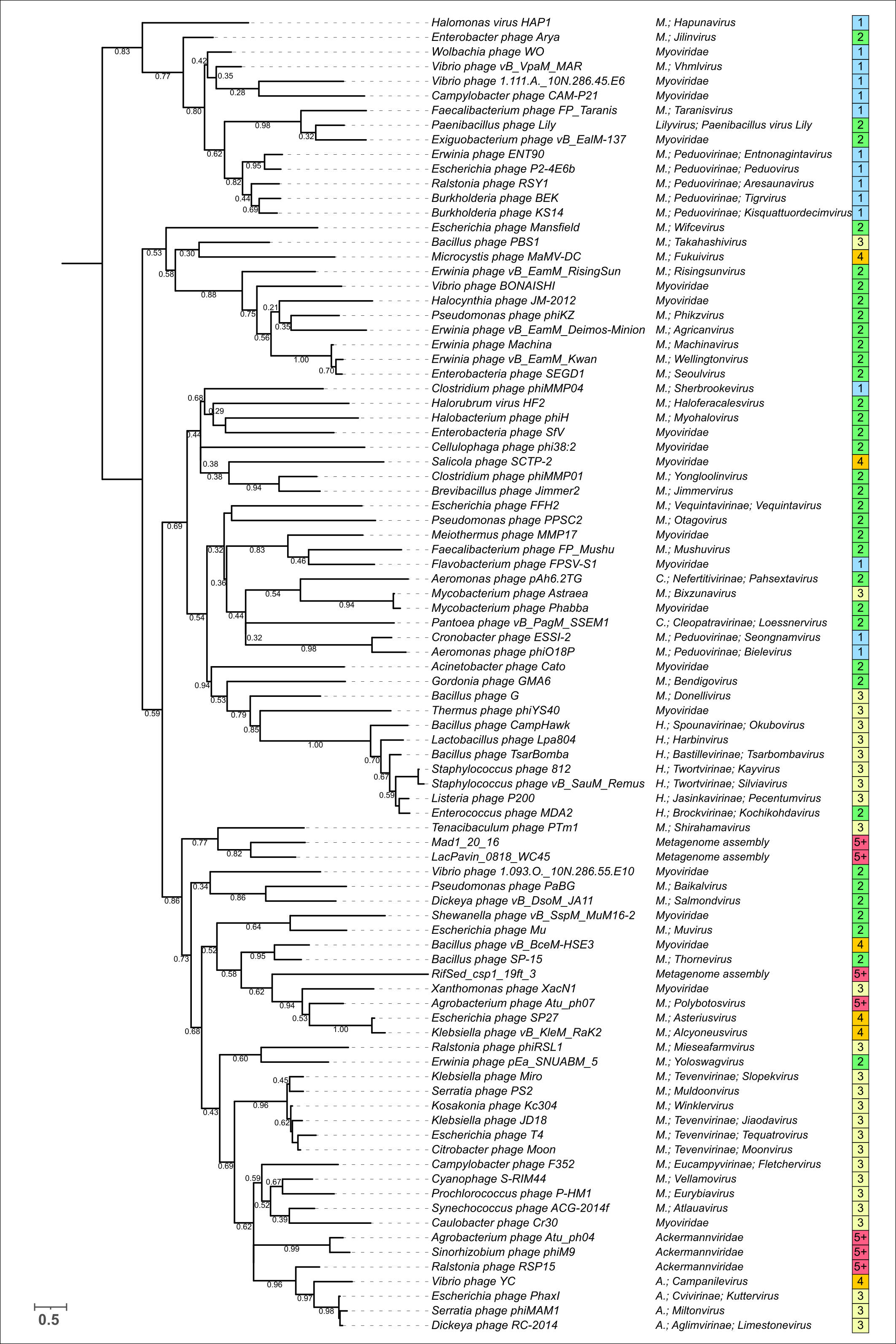

Supplement: Supplementary file 1 [file viruses-14-01148-s001.zip › Supplementary_Figure_S3.jpg]
